# Pathway-driven assessment of wastewater contamination in drinking water systems: integrating AI with public health risk

**DOI:** 10.3389/fpubh.2026.1873246

**Published:** 2026-07-03

**Authors:** Moharana Choudhury, Rohit Kumar, Atin Kumar, Hari Prasad Agarwal, Rakesh Choudhary, Omid Reza Baghchesaraei, Sushobhan Majumdar, Kailash Rajaram Harne, Ajay Kumar

**Affiliations:** 1 Environmental Research and Management Division, Voice of Environment (VoE), Guwahati, Assam, India; 2 Faculty of Agricultural Sciences, GLA University, Mathura, Uttar Pradesh, India; 3 School of Agriculture, Uttaranchal University, Dehradun, Uttarakhand, India; 4 Department of Architecture, The Assam Royal Global University, Guwahati, Assam, India; 5 Department of Civil Engineering, National Institute of Technology Delhi, New Delhi, India; 6 HDR Graduate, Centre for Infrastructure Engineering, Sydney, NSW, Australia; 7 Department of Geography, Pandit Raghunath Murmu Smriti Mahavidyalaya, Bankura, West Bengal, India; 8 Department of Civil Engineering, Netaji Subhas University of Technology, New Delhi, India

**Keywords:** anomaly detection, artificial intelligence, drinking water systems, machine learning, public health risk, sensor-based monitoring, wastewater contamination, water quality monitoring

## Abstract

Wastewater contamination in drinking water systems arises from failures across interconnected components. Globally, an estimated 2.2 billion people lack safely managed drinking water services, with contamination risks persisting in regulated systems. Despite advances in treatment technologies, contamination events continue due to infrastructure deterioration, hydraulic disturbances, and cross-connections, particularly in rapidly urbanizing and resource-constrained regions. A critical limitation of conventional monitoring is its reliance on periodic sampling and laboratory analysis, which often fails to capture transient contamination events and delays response. To address this challenge, artificial intelligence (AI) enables data-driven surveillance through sensor networks, anomaly detection, and predictive modeling. Machine learning and deep learning approaches can identify multivariate contamination patterns, with several studies reporting R^2^ values exceeding 0.70 under controlled conditions. However, most reported performance metrics originate from experimental, pilot-scale, or benchmark datasets with limited long-term operational validation, indicating that predictive success does not necessarily translate into operational readiness. Consequently, real-world applicability remains constrained by data quality limitations, reliance on non-specific proxy indicators, poor model generalizability, and insufficient validation under operational conditions. The absence of explicit linkage between AI-generated outputs and public health response thresholds further limits the translation of detection into actionable risk mitigation. This review synthesizes current understanding of wastewater contamination pathways and critically evaluates both conventional and AI-based monitoring approaches within an integrated engineering–public health framework. Unlike prior reviews emphasizing generic water-quality prediction, this study focuses on pathway-specific contamination detection, system-level vulnerabilities, and operational deployment constraints. It identifies key translational barriers, including sensor reliability, validation gaps, and limited integration into decision-making workflows. The review further distinguishes between predictive performance, evidence readiness, and operational deployment maturity. The analysis highlights that effective implementation of AI requires alignment with risk-based water safety planning and confirmatory monitoring strategies. Future progress must shift from prediction-focused research toward prevention-oriented monitoring systems that enable early detection, reduce exposure duration, and strengthen public health protection. Operational deployment readiness remains substantially less mature than predictive model development. This review proposes a pathway-driven, AI-integrated monitoring framework linking contamination detection to actionable public health response.

## Graphical abstract

**Figure:**
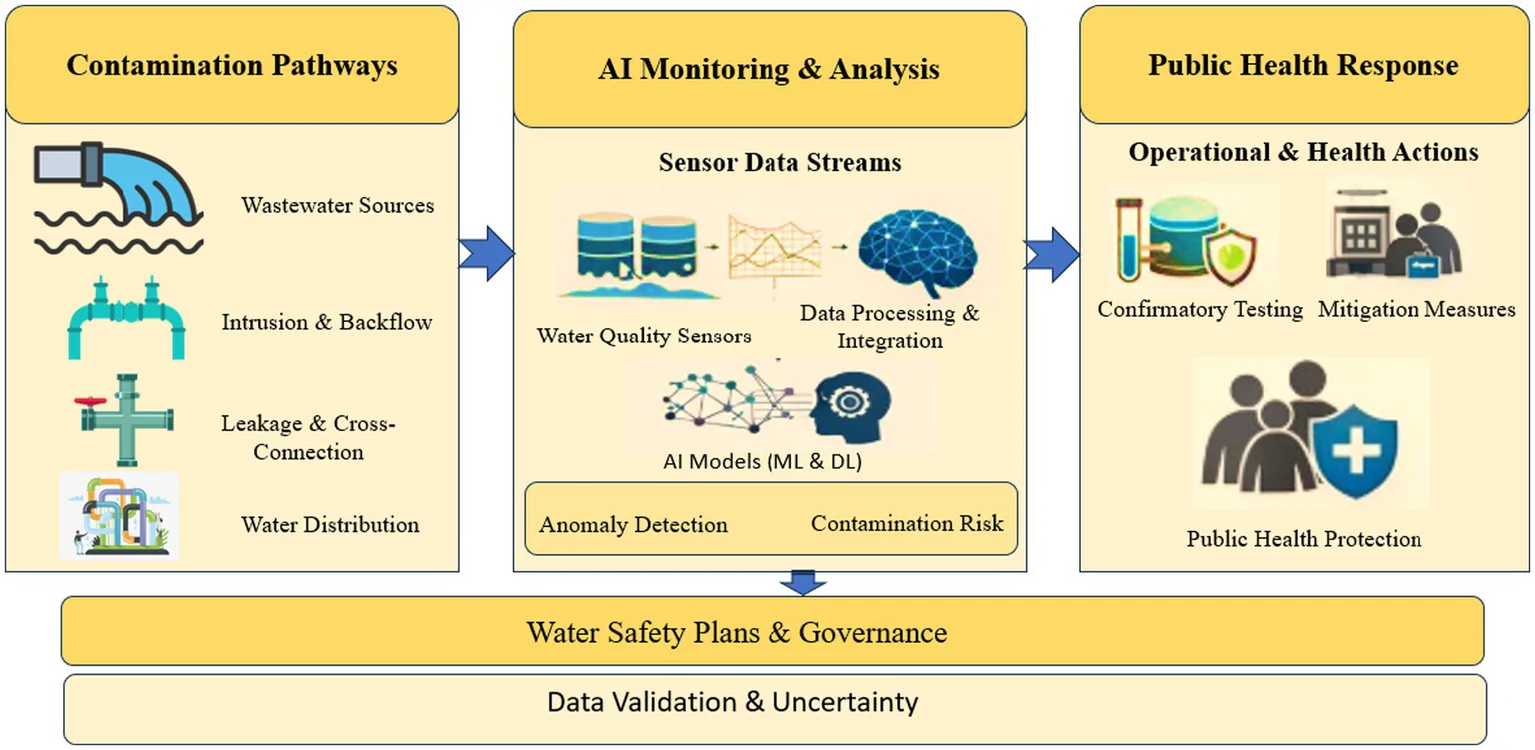

## Highlights

- Wastewater contamination is a pathway-driven, multi-barrier system failure, not a static water quality issue.
- Conventional monitoring fails to detect transient intrusion events due to temporal discontinuity.
- AI enables multi-parameter anomaly detection, but performance is limited by proxy signals and validation bias.
- Sensor uncertainty, validation gaps, and limited operational readiness constrain real-world AI deployment.
- A decision-oriented framework linking AI outputs to public health response thresholds is proposed.

## 1 Introduction

### 1.1 Background and significance

Safe drinking water systems are designed as multi-barrier frameworks to prevent contamination from source to consumption. However, failures across these barriers continue to compromise system integrity under real-world operating conditions. These barriers include source protection, treatment processes, distribution integrity, and premise-level safeguards. Despite advances in treatment technologies, barrier failures persist due to infrastructure aging, hydraulic instability, and operational variability, particularly within complex distribution networks. Recent global assessments indicate that at least 1.7 billion people use drinking water sources contaminated with feces, while 2.2 billion people lack safely managed drinking water services, underscoring the persistence of microbial risks even in regulated systems (1, 2). Rapid urbanization, industrial discharge, and climate variability have further intensified stress on water infrastructure. Climate-driven extreme events such as flooding and drought alter hydraulic conditions, disrupt treatment performance, and increase the likelihood of contamination intrusion and pollutant mobilization within distribution systems (3, 4). Simultaneously, infrastructure deterioration, particularly in low- and middle-income countries (LMICs), exacerbates leakage and pressure instability, creating pathways for external contaminants to enter potable water networks. Wastewater contamination must be conceptualized as a dynamic system-integrity failure governed by interacting physical, hydraulic, and operational breakdowns across the source-to-tap continuum. It emerges when physical, hydraulic, and operational barriers are compromised, allowing contaminated fluids to enter or re-enter drinking water systems. The episodic and short-duration nature of contamination events creates a structural mismatch with conventional monitoring systems, which are inherently incapable of capturing transient intrusion dynamics (5).

### 1.2 Wastewater contamination in drinking water systems as a public health issue

Wastewater intrusion introduces a complex mixture of acute microbial hazards and chronic chemical exposures, linking infrastructure failure directly to population-level health risk. Microbial contamination remains the most immediate concern, with pathogens such as *Escherichia coli*, Campylobacter, and enteric viruses serving as indicators of fecal pollution. Globally, contaminated drinking water is estimated to contribute to approximately 485,000 diarrhoeal deaths annually, with the burden disproportionately affecting low-resource settings (2). Beyond acute microbial risks, wastewater contamination also introduces complex mixtures of chemical pollutants. These include pharmaceuticals, personal care products, endocrine-disrupting compounds, and dissolved organic matter, which can influence disinfection processes and contribute to the formation of harmful by-products. Long-term exposure to such contaminants has been associated with chronic health risks, including carcinogenic and endocrine effects, although these relationships remain incompletely characterized (6). The public health burden is structurally inequitable, with disproportionate exposure risks in LMICs, informal settlements, and intermittently supplied systems. Populations in LMICs, informal settlements, and peri-urban regions face disproportionately higher exposure risks due to intermittent water supply, inadequate sanitation infrastructure, and limited monitoring capacity. However, even in high-income countries, distribution system failures have been linked to localized outbreaks, indicating that advanced treatment does not eliminate downstream vulnerabilities (7). Exposure pathways extend beyond direct ingestion. Domestic uses such as food preparation, hygiene, and water storage can amplify exposure, particularly in households lacking safe handling practices. This highlights the need to integrate engineering-based monitoring with public health surveillance systems to enable timely detection and exposure mitigation.

### 1.3 Why AI-based approaches are gaining attention

Conventional water quality monitoring relies heavily on periodic sampling and laboratory-based analysis. While these methods provide high analytical accuracy, they suffer from temporal discontinuity and delayed reporting. Contamination events, particularly those driven by transient hydraulic disturbances, may occur within hours, whereas laboratory confirmation often requires days. This temporal mismatch limits the ability of utilities to respond proactively. The inability of conventional monitoring to capture transient contamination events has driven the adoption of real-time sensor data and digital monitoring platforms, creating opportunities for artificial intelligence (AI)-based analysis. Machine learning (ML) models can process multivariate data streams to detect contamination-specific signatures associated with wastewater intrusion, rather than general water quality variation. These capabilities are essential for wastewater contamination detection, where signals are often subtle and embedded within complex background variability (8). AI-based approaches have been applied to tasks such as contamination detection, microbial risk prediction, and anomaly identification within distribution systems. Field deployments integrating fluorescence sensors with ML classifiers have demonstrated near real-time fecal contamination detection in operational drinking water systems. For instance, models integrating fluorescence sensor data with ML algorithms have demonstrated the ability to classify fecal contamination risk in near real time, offering a potential alternative to time-intensive microbiological assays (9). However, their effectiveness remains constrained by data quality, sensor reliability, and inadequate real-world validation. Additionally, limited integration with operational decision frameworks reduces their practical impact on contamination response. Thus, while AI offers significant potential for enhancing monitoring capabilities, its effectiveness is contingent upon integration with robust sensing infrastructure and governance frameworks.

### 1.4 Knowledge gap and rationale for the review

Despite growing interest in AI-based water quality monitoring, existing literature remains fragmented, with a dominant focus on predictive model performance rather than contamination pathway specificity, operational deployment constraints, and public health translation. It also lacks detailed pathway-specific analysis of wastewater contamination in drinking water systems. Existing reviews often focus on general water quality prediction without distinguishing between contamination sources or system-specific dynamics. Others emphasize algorithmic development while overlooking practical deployment challenges and public health implications. A critical limitation in current research is the lack of pathway-specific analysis. Many studies rely on surrogate parameters such as turbidity or conductivity, which lack specificity for wastewater contamination and increase the risk of misclassification. Future approaches should prioritize integration of multi-signal indicators, including fluorescence-based organic signatures, microbial genetic markers, and hydraulic context variables, to improve specificity in wastewater contamination detection. This reliance on non-specific surrogate indicators introduces signal ambiguity, increasing both false-positive detection and critical false-negative risk under real-world conditions. Furthermore, model performance is frequently evaluated using random train-test splits, which ignore temporal dependencies and overestimate predictive reliability under real-world conditions (5). There is also limited integration between contamination detection and public health risk assessment. While wastewater-based epidemiology has demonstrated the value of environmental monitoring for disease surveillance, its application within drinking water systems remains underdeveloped. Consequently, a comprehensive synthesis linking contamination pathways, AI-based detection, and health outcomes is lacking.

### 1.5 Objectives and scope

**Figure 1:**
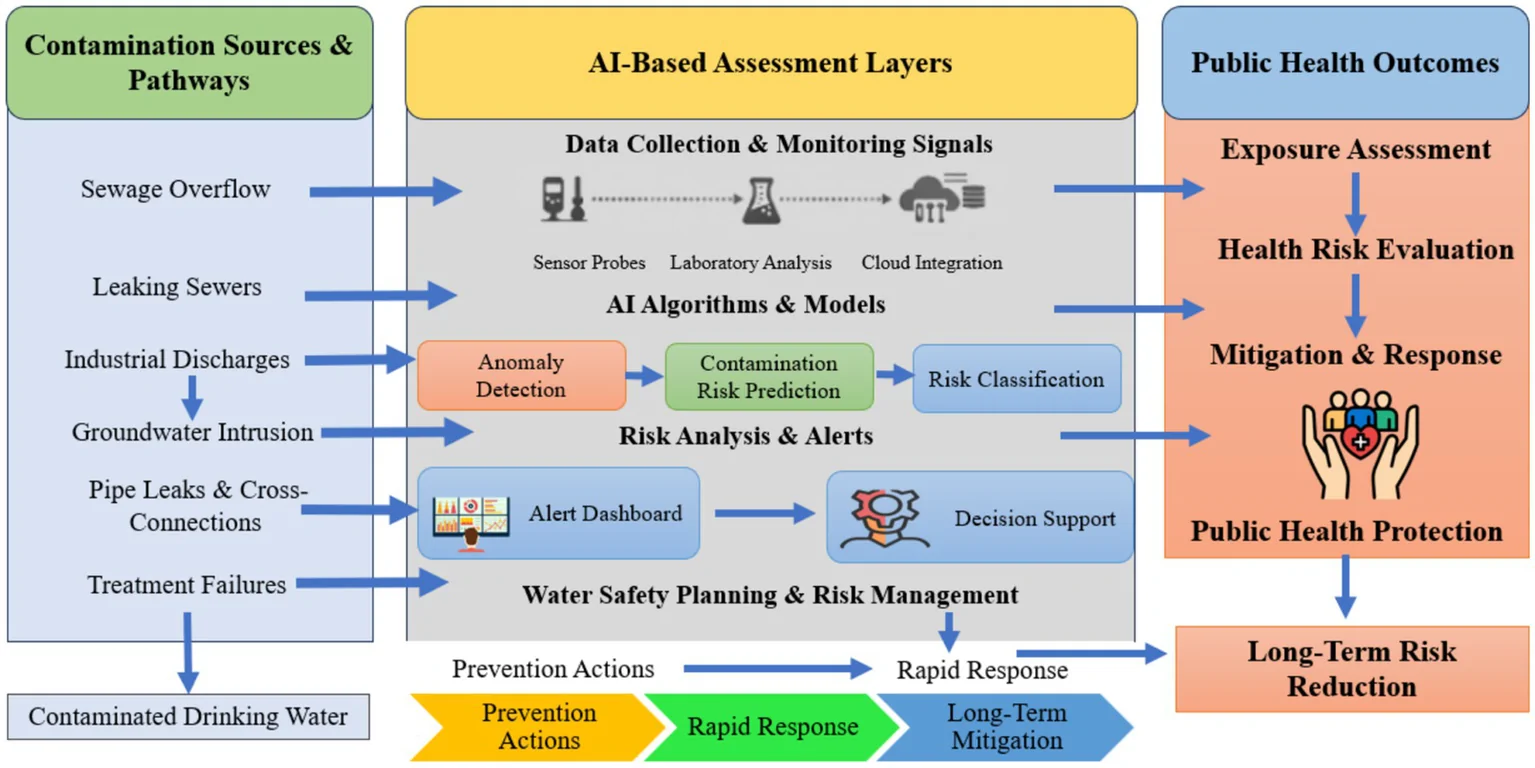
Pathway-driven conceptual framework illustrating the linkage between wastewater contamination sources, AI-enabled assessment layers, operational decision-support systems, and public health response outcomes in drinking water systems. Developed by the authors.

This review provides a structured and critical synthesis of wastewater contamination in drinking water systems, with a focus on AI-based assessment approaches and their implications for public health. Specifically, it evaluates contamination sources and pathways, compares conventional and AI-driven detection methods, and assesses their applicability within real-world operational contexts. The novelty of this review lies in its pathway-driven synthesis of wastewater contamination in drinking water systems, where AI-based monitoring is evaluated not only as a predictive tool but also as part of a broader exposure-risk and public health response pathway. Existing reviews have primarily focused on machine-learning applications for general water-quality prediction, algorithmic performance benchmarking, or smart monitoring technologies. While these contributions have advanced understanding of AI methodologies, they often provide limited discussion of wastewater-specific contamination pathways, operational deployment constraints, public health interpretation, and decision-oriented response mechanisms. In contrast, the present review adopts a pathway-driven perspective that explicitly links contamination sources, monitoring signals, AI-based detection, validation limitations, operational readiness, and public health response within a unified source-to-tap risk-management framework. This integrated perspective addresses an important translational gap between predictive model development and practical drinking-water safety management. Unlike previous reviews that primarily emphasize generic water-quality prediction or algorithmic performance, this review critically distinguishes between established evidence, unresolved uncertainties, and translational barriers. Established evidence shows that sensor-enabled monitoring and machine-learning models can improve detection sensitivity and temporal responsiveness of abnormal water-quality patterns under controlled or well-characterized conditions. However, uncertainty remains regarding the specificity of proxy indicators, the transferability of models across distribution systems, performance under low-frequency and transient contamination events, and integration with confirmatory testing and public health decision thresholds. Therefore, the contribution of this review is not limited to proposing a conceptual framework; it also evaluates the operational readiness of AI-enabled monitoring by linking contamination pathways, validation limitations, deployment constraints, and public health response requirements within a unified source-to-tap risk-management and decision-support perspective. The scope is limited to drinking water systems, including source water, treatment processes, distribution networks, and premise plumbing. Studies focusing solely on surface water monitoring, remote sensing, or spatial analysis without direct relevance to contamination pathways are excluded. Figure 1 illustrates the conceptual framework linking wastewater contamination pathways with AI-enabled monitoring and public health outcomes. Collectively, the review is structured to examine contamination pathways, monitoring approaches, AI-enabled analytical frameworks, and public health response systems within an integrated source-to-tap risk-management perspective.

The framework highlights how failures in system barriers generate measurable signals, which can be interpreted through AI-based analytical layers to support early detection and decision-making. It also emphasizes the critical link between engineering monitoring and public health protection, forming the basis for the subsequent analysis. Importantly, the framework emphasizes that AI outputs should be interpreted within operational and public health decision contexts rather than as standalone indicators of contamination events.

## 2 Review scope, literature framing, and analytical approach

### 2.1 Scope of the review

This review focuses specifically on wastewater contamination within drinking water systems, treating it as a pathway-driven and system-integrity problem rather than a general water quality issue. The analytical boundary extends across the full drinking water chain, including source water, treatment processes, distribution systems, and premise plumbing, where contamination can occur through intrusion, cross-connections, or operational failures (10). The review prioritizes contamination events linked to wastewater-derived signals, including microbial indicators, organic matter shifts, and chemical tracers associated with sewage inputs. Studies that examine generic water quality prediction without distinguishing contamination sources are considered only where they contribute methodological insight into detection or modeling approaches. Exclusions are intentionally defined to maintain analytical clarity. Research focused solely on surface water monitoring, remote sensing, or spatial distribution analysis is not included unless it directly informs contamination pathways or drinking water system vulnerability. Similarly, purely algorithmic studies that do not demonstrate relevance to environmental monitoring or public health interpretation are excluded. This boundary ensures that the review remains focused on operationally meaningful and health-relevant contamination assessment.

### 2.2 Thematic organization

The structure of this review follows a dual analytical spine. The first spine addresses contamination pathways, including sources, entry mechanisms, and determinants of severity. The second spine focuses on assessment and detection frameworks, comparing conventional monitoring approaches with AI-based methods. These two strands converge in later sections that evaluate public health implications, translational barriers, and governance considerations. This thematic organization is designed to move from system understanding to methodological evaluation and finally to decision relevance. Early sections establish how contamination occurs and why it is difficult to detect. Subsequent sections assess how existing tools attempt to address these challenges, highlighting both strengths and limitations. The final sections synthesize these insights into a framework for improving monitoring, risk management, and public health protection. By structuring the review in this way, the analysis avoids fragmented discussion of methods or contaminants in isolation. Instead, it emphasizes the interdependence between system behavior, detection capability, and health outcomes, which is essential for a critical evaluation of AI-based approaches.

### 2.3 Analytical approach

This study follows a structured critical review approach that combines systematic literature identification, qualitative synthesis, and methodological appraisal, guided by the PRISMA 2020 (Preferred Reporting Items for Systematic Reviews and Meta-Analyses) framework. The literature search was carried out exclusively using the Scopus database, chosen for its extensive coverage of peer-reviewed research in environmental science, engineering, and AI, along with its consistent indexing of abstracts, keywords, and citation metadata that supports transparent and reproducible analysis. Although additional databases may contain relevant studies, Scopus was selected as the primary source because of its broad multidisciplinary coverage, standardized indexing structure, and suitability for reproducible bibliometric and thematic screening.

**Figure 2:**
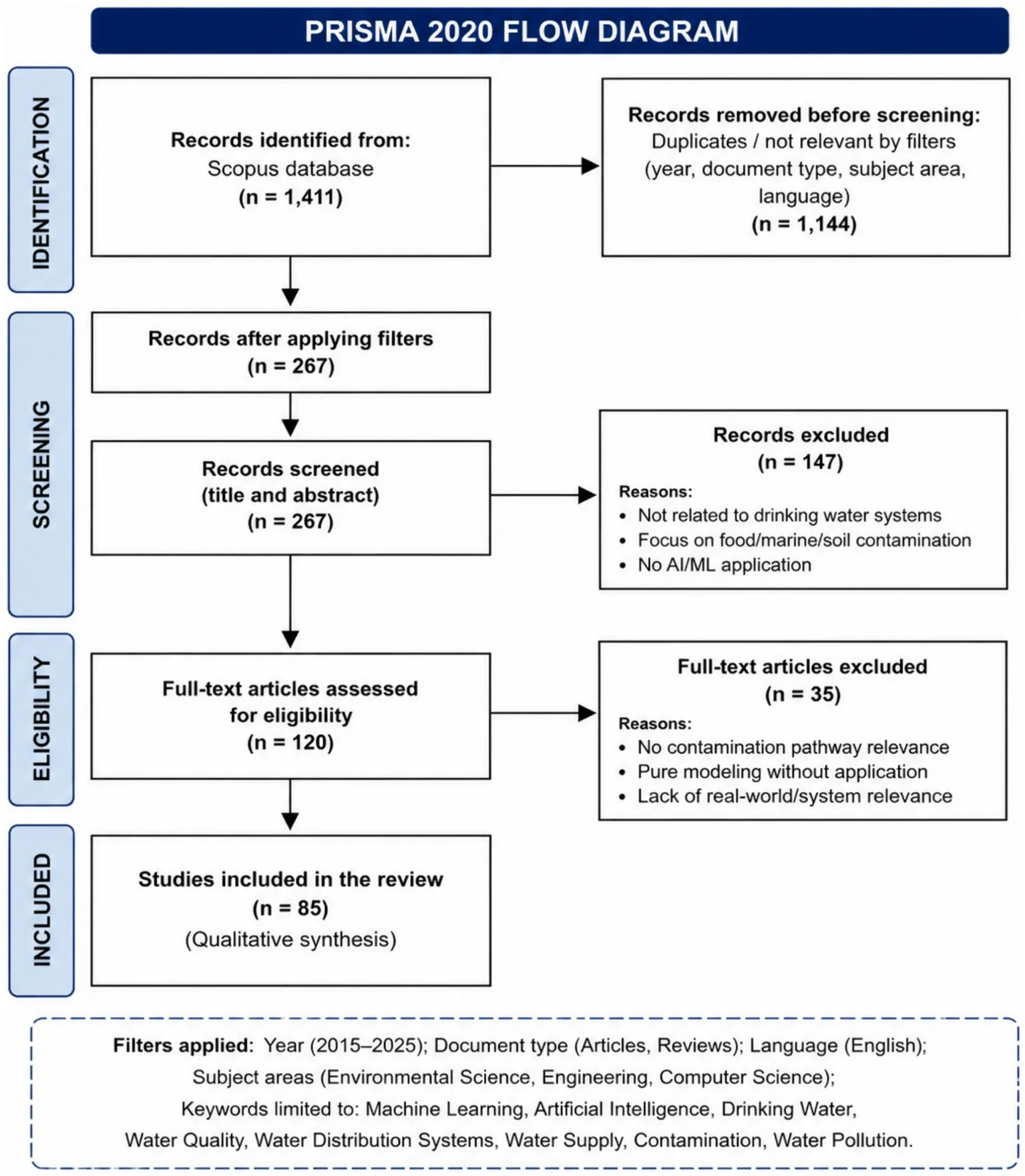
PRISMA flow diagram of the study selection process using the Scopus database. Developed by the authors following the PRISMA 2020 guidelines.

The search strategy was developed using combinations of keywords such as “drinking water contamination,” “water contamination,” “wastewater intrusion,” “ML,” and “water quality monitoring,” applied across article titles, abstracts, and keywords. The literature search was conducted in the Scopus database on 05 May 2026 using TITLE-ABS-KEY search fields to capture relevant studies from titles, abstracts, and author keywords. The primary search string included combinations of terms related to drinking water contamination, wastewater intrusion, AI, ML, anomaly detection, and water quality monitoring. A representative search query was structured as follows: TITLE-ABS-KEY [(“drinking water contamination” OR “wastewater intrusion” OR “water contamination”) AND (“ML” OR “AI” OR “deep learning (DL)” OR “anomaly detection”) AND (“water quality monitoring” OR “sensor-based monitoring”)]. The retrieved studies were screened manually through title, abstract, and full-text evaluation. The screening and eligibility assessment process was conducted independently by the authors, and disagreements regarding study inclusion were resolved through discussion and consensus. To improve selection consistency, eligibility assessment focused specifically on studies addressing contamination pathways, AI-enabled monitoring, operational applicability, and public health interpretation within drinking water systems. Although Scopus was selected because of its broad interdisciplinary indexing across environmental science, engineering, and AI-related literature, the exclusion of additional databases such as Web of Science and PubMed/MEDLINE may have limited the comprehensiveness of the evidence base, particularly for epidemiological and public health-oriented studies. This initial search returned 1,411 records. To focus the dataset, predefined filters were applied, including publication years (2015–2025), document types (articles and reviews), language (English), and subject areas (Environmental Science, Engineering, and Computer Science). After applying these filters, 267 records were retained for further evaluation. The screening process was conducted in two stages. The screening and eligibility assessment process was conducted independently by multiple authors, and disagreements regarding study inclusion were resolved through discussion and consensus. First, titles and abstracts of the 267 records were reviewed to assess their relevance to drinking water systems and AI-based monitoring. At this stage, 147 records were excluded, primarily because they addressed unrelated domains such as food, marine, or soil contamination, or did not incorporate ML or AI approaches. The remaining 120 articles were then examined in full text against predefined eligibility criteria, including relevance to wastewater contamination pathways in drinking water systems, application of AI and ML techniques, and contribution to environmental monitoring or public health interpretation. During this stage, 35 studies were excluded due to limited system relevance, lack of methodological clarity, or absence of practical application. As a result, 85 studies were selected for the final qualitative synthesis. The study selection workflow is presented in Figure 2.

The PRISMA-based process provides a clear and traceable record of how studies were identified, filtered, and selected, helping to minimize bias and improve transparency throughout the review. The evidence base was organized into three complementary groups: review articles that support conceptual understanding, empirical studies that provide field-based or operational insights, and public health or policy-oriented studies that help interpret broader implications. This combination allows the analysis to remain grounded in both methodological developments and practical applications. Studies were examined using a set of predefined appraisal criteria, including data quality, validation approach, interpretability of models, operational feasibility, and relevance to public health decision-making. Particular attention was given to recurring issues in AI-based environmental studies, such as dependence on proxy variables, limited temporal or external validation, and lack of real-world deployment. These aspects are important when considering whether reported model performance can be relied upon in practical monitoring scenarios (11). The criteria used to define the review scope, inclusion boundaries, and evaluation approach are summarized in Table 1.

**Table 1:** Analytical framework and inclusion–exclusion criteria for evaluating literature on wastewater contamination and AI-based monitoring in drinking water systems.

Review domain	Included focus	Excluded focus	Rationale	Appraisal criteria	References
Drinking water systems	Source–treatment–distribution–premise plumbing continuum	Surface water-only studies	Focus on exposure pathway	System relevance, contamination linkage	(10)
Wastewater contamination	Microbial, chemical, tracer-based contamination	Generic water quality variation	Specificity of contamination	Signal specificity, health relevance	(5)
Detection methods	Conventional + AI-based monitoring	Purely theoretical modeling	Operational applicability	Validation design, uncertainty reporting	(8)
AI approaches	ML, DL, hybrid models for prediction/detection	Algorithm-only studies	Translational relevance	External validation, interpretability	(11)
Public health linkage	Exposure pathways and risk interpretation	Engineering-only focus	Health relevance	Exposure–risk linkage, policy relevance	(1)
Monitoring systems	Continuous sensing, IoT-based systems	Remote sensing-only studies	System-level applicability	Deployment feasibility, scalability	(9)
Contaminants	Heavy metals, pathogens, nutrients	Food and marine contamination	Drinking water focus	Regulatory and health significance	(1)
Study design	Empirical, applied, and review studies	Editorials, abstracts	Methodological rigor	Data quality, reproducibility	–
Timeframe	2015–2025	Pre-2015 studies	Recent advancements	Relevance and innovation	–
Language	English	Non-English	Consistency	Clarity and completeness	–

Compiled and synthesized by the authors.

The framework provides a consistent basis for comparing studies across different approaches and disciplines, while making the selection logic explicit. It also supports a more transparent interpretation of results by clearly linking each study to defined evaluation criteria, which is particularly important given the diversity of methods and applications within this research area. This review has certain limitations. The literature search was restricted to English-language publications indexed in the Scopus database between 2015 and 2025. Consequently, relevant studies indexed elsewhere or published in non-English sources may not have been captured. In addition, the rapidly evolving nature of AI research means that newly emerging methodologies may not yet be fully represented.

## 3 Sources, types, and pathways of wastewater contamination in drinking water systems

### 3.1 Major sources

Wastewater contamination in drinking water systems arises from multiple interacting sources, forming a coupled environmental–infrastructure system with both direct and indirect intrusion pathways. These pathways reflect failures in sanitation infrastructure, environmental management, and system design. Municipal wastewater remains the dominant contributor, particularly in urban regions where sewer networks operate under high hydraulic loads and aging conditions. Sewer leakage and exfiltration have been identified as significant contributors to environmental contamination, allowing untreated wastewater to infiltrate surrounding soil and groundwater systems, which may later interact with drinking water infrastructure (3). Industrial discharges further complicate contamination profiles by introducing heavy metals, organic solvents, and complex chemical mixtures into wastewater streams. These contaminants may persist through treatment processes or accumulate in environmental reservoirs, increasing the likelihood of indirect entry into drinking water systems. In many LMIC contexts, inadequate regulation of industrial effluents exacerbates this risk, leading to co-contamination scenarios where microbial and chemical hazards coexist (6). On-site sanitation systems, including septic tanks and pit latrines, represent another critical source, particularly in peri-urban and rural settings. Poorly designed or maintained systems can leak into shallow groundwater, which is often used as a drinking water source. Studies have shown that proximity between sanitation facilities and water supply points significantly increases the probability of fecal contamination, especially under conditions of high population density and limited infrastructure planning (1). Agricultural activities also contribute indirectly through runoff containing nutrients, pathogens, and organic matter derived from livestock waste. While typically associated with surface water contamination, these inputs can infiltrate groundwater or interact with distribution systems under certain hydraulic conditions. The convergence of these sources creates a complex contamination landscape, where distinguishing wastewater-derived signals becomes analytically challenging.

### 3.2 Modes of entry into drinking water systems

**Figure 3:**
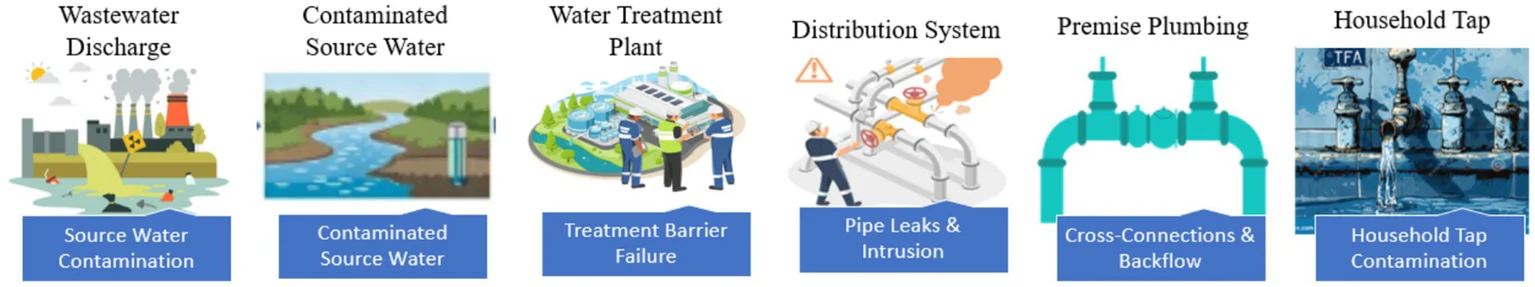
Conceptual representation of wastewater contamination pathways and barrier failure dynamics across drinking water infrastructure, highlighting intrusion mechanisms, hydraulic vulnerability, and operational failure points. Developed by the authors based on the reviewed literature.

The transition from contamination source to drinking water exposure is governed by a series of entry mechanisms that reflect system vulnerabilities. Among these, cross-connections and backflow events are widely recognized as primary pathways for wastewater intrusion. Cross-connections occur when potable water systems are inadvertently connected to non-potable sources, allowing contaminated water to enter under conditions of pressure imbalance (12). Pressure transients within distribution networks represent another critical mechanism. Sudden drops in pressure, often caused by pipe breaks, pump failures, or intermittent supply, can create negative pressure zones that draw contaminated external fluids into the system. These intrusion events are typically short-lived but can introduce significant contaminant loads, particularly when occurring near sources of wastewater contamination (3). Leakage and pipe deterioration further exacerbate intrusion risk. Aging infrastructure, characterized by cracks, corrosion, and joint failures, provides physical pathways for contaminant entry. The likelihood of intrusion increases in systems with high leakage rates, where external water can infiltrate the network under fluctuating hydraulic conditions (5). Storage and distribution components, including reservoirs and tanks, also serve as potential points of contamination. Inadequate maintenance, structural defects, or exposure to environmental contaminants can compromise water quality at these stages. Additionally, premise plumbing systems, particularly in buildings with complex internal networks, can introduce contamination through backflow, stagnation, or improper connections. These entry mechanisms highlight that contamination is not confined to a single point but can occur throughout the distribution chain. Effective monitoring therefore requires a system-wide perspective that accounts for both structural vulnerabilities and operational dynamics. Figure 3 illustrates the major pathways through which wastewater contamination can enter drinking water systems, highlighting key failure points across the source-to-tap continuum.

Figure 3 emphasizes that contamination pathways are governed by both physical infrastructure conditions and hydraulic dynamics, reinforcing the need for integrated monitoring strategies that capture these interactions.

### 3.3 Types of contaminants

Wastewater contamination introduces a diverse range of contaminants into drinking water systems, broadly categorized into microbial, chemical, and emerging hazard classes. Microbial contaminants remain the most immediate concern due to their direct association with fecal pollution and disease transmission. Indicators such as *Escherichia coli* and enterococci are widely used to signal fecal contamination, while pathogens including viruses and protozoa represent direct health risks (1). Opportunistic pathogens, such as Legionella and Pseudomonas, also play a significant role, particularly within distribution systems where biofilms provide favorable growth environments. These organisms may not originate directly from wastewater but can proliferate following contamination events that alter system conditions (10). Chemical contaminants associated with wastewater include nutrients, pharmaceuticals, personal care products, and dissolved organic matter. These substances can influence treatment processes and contribute to the formation of disinfection by-products, which have been linked to long-term health risks. Additionally, wastewater-derived organic matter can alter the physicochemical properties of water, affecting both treatment efficiency and distribution system stability (6). Emerging concerns include antimicrobial resistance genes (ARGs), which can be transported through wastewater and persist within drinking water systems. The presence of ARGs raises concerns about the potential spread of antibiotic resistance through environmental pathways, although the extent of this risk remains under investigation (10). The diversity of contaminants underscores the complexity of wastewater contamination, requiring detection approaches that can capture both specific and non-specific signals.

### 3.4 Determinants of contamination severity

The impact of wastewater contamination on drinking water systems is influenced by a combination of physical, chemical, and operational factors. Hydraulic conditions play a central role, with pressure fluctuations, flow velocity, and residence time affecting both the likelihood of intrusion and the distribution of contaminants within the system. Residual disinfectant levels are critical in determining microbial survival. Systems with inadequate disinfectant residuals are more susceptible to pathogen persistence and regrowth, particularly following contamination events. Conversely, high disinfectant levels may mitigate microbial risks but can also contribute to the formation of harmful by-products in the presence of organic contaminants (13). Biofilm dynamics within distribution systems further influence contamination outcomes. Biofilms can act as reservoirs for both microorganisms and chemical contaminants, facilitating their persistence and potential release into the bulk water. Changes in hydraulic or chemical conditions can destabilize biofilms, leading to episodic contamination events (14). Environmental factors, including temperature and seasonal variability, also affect contamination processes. Higher temperatures can enhance microbial growth and chemical reactions, while extreme weather events can increase the likelihood of system disruptions and contamination intrusion (10). In systems with delayed detection, even short-duration contamination events can result in significant exposure, particularly for microbial hazards with low infectious doses. Delays in detection and response can significantly increase exposure risk, particularly for microbial contaminants with rapid transmission potential. This highlights the importance of real-time or rapid contamination detection system capable of capturing transient contamination events. Table 2 provides a comprehensive overview of wastewater-derived contaminants, their sources, entry mechanisms, and associated public health relevance.

**Table 2:** Wastewater-derived contaminants, indicators, tracers, and associated public health relevance in drinking water systems.

Contaminant class	Example indicators/Tracers	Major source	Mode of entry	Health relevance	Measurement approach	Signal specificity	References
Microbial indicators	*E. coli*, Enterococci	Sewage, septic leakage	Intrusion, cross-connection	Acute gastrointestinal disease	Culture-based methods	High	(1)
Pathogens	Viruses, Protozoa	Wastewater discharge	Pressure transients	Infectious disease outbreaks	Molecular assays (qPCR)	High	(25)
Opportunistic pathogens	Legionella	Biofilms	System regrowth	Respiratory infections	Culture/PCR	Moderate	(26)
Nutrients	Nitrate, Phosphate	Agricultural runoff	Source contamination	Indirect health effects	Spectroscopy	Low	(27)
Organic contaminants	Pharmaceuticals	Wastewater effluent	Leakage, intrusion	Chronic toxicity	Chromatography	Moderate	(28)
Organic matter	DOC, Fluorescence	Sewage intrusion	Distribution system	DBP formation risk	Optical sensors	Moderate	(29)
ARGs	Resistance genes	Wastewater	Intrusion	Antibiotic resistance spread	Molecular methods	Emerging	(30)

Compiled and synthesized by the authors from the reviewed literature.

The table demonstrates that wastewater contamination cannot be captured through a single indicator or measurement approach. Instead, it requires a combination of microbial, chemical, and surrogate signals, each with varying levels of specificity and operational feasibility. This complexity directly informs the need for advanced detection and analytical approaches, which are examined in the following sections.

## 4 Conventional approaches for detecting and assessing wastewater contamination

### 4.1 Physicochemical indicators

Conventional assessment of wastewater contamination in drinking water systems relies heavily on physicochemical indicators that act as indirect signals of water quality alteration. Parameters such as turbidity, electrical conductivity, pH, dissolved oxygen, and residual chlorine are routinely monitored due to their operational simplicity and real-time measurability. Among these, turbidity increases are often associated with particulate intrusion, while conductivity changes may indicate the mixing of external water sources, including wastewater-derived inputs (15). Residual chlorine plays a particularly important role as both a treatment indicator and a contamination proxy. A sudden decline in disinfectant residual can signal increased organic load or microbial activity, both of which are consistent with wastewater intrusion events. However, such signals are inherently non-specific, as chlorine decay may also result from natural organic matter fluctuations or operational adjustments in treatment systems (10). Parameters such as total organic carbon, dissolved organic carbon, and UV254 absorbance provide further insight into organic contamination. Wastewater-derived organic matter typically exhibits distinct optical properties, allowing these indicators to function as surrogate markers. However, the overlap between natural and anthropogenic organic signals limits their ability to definitively attribute contamination to wastewater sources (16). Nutrient concentrations, including nitrate and phosphate, are also frequently monitored. While elevated nutrient levels may indicate contamination, they are influenced by multiple environmental and anthropogenic factors, reducing their diagnostic specificity. This highlights a fundamental limitation of physicochemical indicators: they are effective for detecting anomalies, but not for confirming contamination origin.

### 4.2 Microbial and biological indicators

Microbial indicators remain the cornerstone of wastewater contamination assessment due to their direct linkage with fecal pollution and public health risk. Among these, *Escherichia coli* and total coliforms are widely used as primary indicators of fecal contamination in drinking water systems. Their presence suggests potential exposure to pathogenic microorganisms, including viruses, bacteria, and protozoa (1). Culture-based methods for detecting microbial indicators are well-established and standardized, making them suitable for regulatory compliance and long-term monitoring. However, these methods are inherently time-consuming, often requiring 18–48 h to produce results. This delay creates a critical gap between contamination occurrence and detection, during which exposure may already have occurred (7). Enterococci and other fecal indicator bacteria provide complementary information, particularly in systems where *E. coli* detection may be inconsistent. Additionally, microbial indicators are increasingly being supplemented with molecular techniques that can detect specific pathogens or genetic markers. However, even these approaches face challenges related to cost, technical complexity, and interpretation of results in dynamic environmental conditions (10). Biological indicators are highly relevant for confirming contamination but are less effective for early warning. Their utility lies in validation rather than prediction, reinforcing the need for complementary monitoring approaches (17).

### 4.3 Instrumental and laboratory methods

Advanced instrumental and laboratory techniques have expanded the capability to detect and characterize wastewater contamination with greater sensitivity and specificity. Molecular methods such as quantitative polymerase chain reaction (qPCR) and digital PCR (dPCR) enable rapid detection of microbial DNA, including pathogens and ARGs. These techniques provide high-resolution insights into contamination profiles but require specialized infrastructure and expertise (10). Spectroscopic methods, including fluorescence spectroscopy, have gained attention as potential rapid-response indicator of wastewater-derived organic matter. Fluorescence signatures associated with protein-like and humic-like substances can serve as proxies for fecal contamination. When combined with ML models, these signals have demonstrated potential for near-real-time classification of contamination risk levels (9). Chromatographic techniques, such as gas chromatography–mass spectrometry (GC–MS) and liquid chromatography–mass spectrometry (LC–MS), are used to detect trace organic contaminants, including pharmaceuticals and personal care products. These methods provide high specificity but are limited by cost, complexity, and lack of real-time capability (6). Metagenomic approaches offer a comprehensive view of microbial communities within water systems, enabling identification of both known and emerging pathogens. However, their application in routine monitoring remains limited due to high costs and challenges in data interpretation. These instrumental methods are essential for confirmatory analysis but are not designed for continuous monitoring or rapid response, highlighting their role within a broader surveillance framework rather than as standalone solutions.

### 4.4 Limitations of conventional assessment

**Figure 4:**
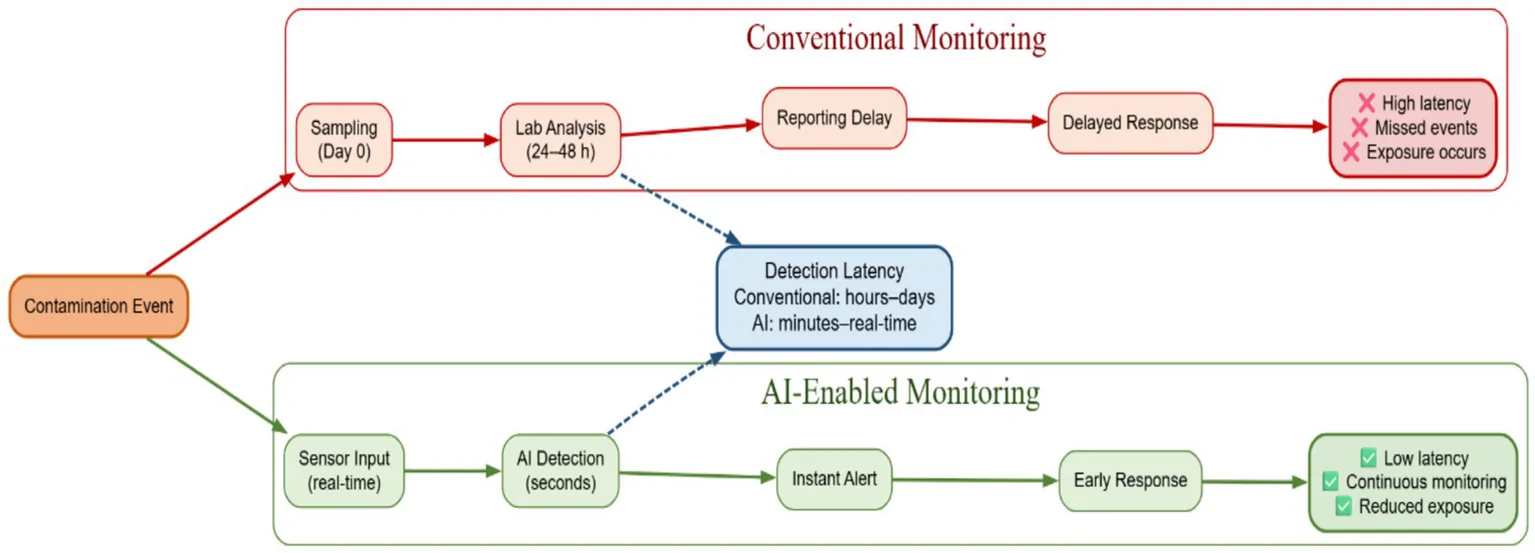
Detection timeline comparison between conventional monitoring and AI-enabled surveillance for wastewater contamination events in drinking water systems. Developed by the authors based on the reviewed literature.

Despite their widespread use, conventional approaches exhibit several critical limitations when applied to wastewater contamination detection in drinking water systems. The most significant limitation is temporal mismatch, where sampling frequency is insufficient to capture transient contamination events. Intrusion events often occur over short time scales, while conventional monitoring operates on daily or weekly intervals, leading to potential under-detection (3). Another limitation is the lack of specificity in many commonly used indicators. Physicochemical parameters can signal changes in water quality but cannot reliably distinguish between wastewater contamination and other sources of variation. This creates uncertainty in interpretation and may result in false alarms or missed events (5). Missed events (false negatives) are particularly critical, as they directly translate into undetected exposure and increased public health risk. Operational constraints further limit the effectiveness of conventional methods. Laboratory-based analyses require significant resources, including trained personnel, equipment, and time. In many settings, particularly in LMICs, these requirements restrict the frequency and scope of monitoring, increasing vulnerability to undetected contamination events (1). To clarify this temporal limitation, Figure 4 compares the detection timeline of conventional monitoring with AI-enabled surveillance during a contamination event.

Conventional monitoring depends on sampling, laboratory analysis, and reporting, which can delay response from hours to days. AI-enabled monitoring integrates real-time sensor inputs, anomaly detection, and automated alerts, allowing earlier response and reduced exposure risk. They provide retrospective confirmation of water quality but do not support proactive detection or early warning. This limitation is particularly critical in the context of public health, where timely intervention is essential to prevent exposure and disease transmission. Table 3 summarizes the major conventional methods used for wastewater contamination assessment, highlighting their strengths, limitations, and suitability for different monitoring objectives.

**Table 3:** Conventional methods for wastewater contamination assessment in drinking water systems: analytical strengths, limitations, and operational suitability.

Method type	Target signal	Turn-around time	Strength	Key limitation	Early warning suitability	Confirmation suitability	References
Turbidity measurement	Suspended particles	Real-time	Simple, continuous monitoring	Non-specific	High	Low	(31)
Conductivity	Ionic changes	Real-time	Sensitive to mixing	Low specificity	Moderate	Low	(32)
Residual chlorine	Disinfection status	Real-time	Indicates microbial risk indirectly	Influenced by multiple factors	Moderate	Low	(33)
TOC/DOC	Organic matter	Hours	Indicates organic load	Cannot confirm source	Moderate	Low	(34)
*E. coli* culture	Fecal contamination	24–48 h	High relevance to health	Slow detection	Low	High	(35)
qPCR	Pathogen DNA	Hours	High sensitivity	Cost and complexity	Moderate	High	(36)
Fluorescence spectroscopy	Organic signatures	Near real-time	Proxy for wastewater signals	Requires calibration	High	Moderate	(37)
GC–MS/LC–MS	Trace chemicals	Days	High specificity	Expensive, non-real-time	Low	High	(38)

Compiled and synthesized by the authors from the reviewed literature.

The table reinforces that no single conventional method can provide comprehensive detection of wastewater contamination. Instead, effective assessment requires a combination of indicators and techniques, each addressing different aspects of the contamination process. This limitation directly motivates the integration of advanced analytical approaches, particularly AI-based methods, which aim to enhance detection capability, improve temporal resolution, and support decision-making. The following section critically examines these approaches and their applicability in real-world drinking water systems.

## 5 AI-based approaches for contamination detection, prediction, and decision support

### 5.1 Relevance of AI in this domain

The increasing complexity of drinking water systems, coupled with the episodic and transient nature of wastewater contamination, has exposed fundamental limitations of conventional monitoring approaches. Contamination events may initially appear as low-amplitude perturbations embedded within normal operational variability, requiring multivariate and temporal analysis for reliable detection rather than simple threshold exceedance methods (42). This has led to the adoption of AI as a tool for extracting hidden patterns, detecting anomalies, and supporting early warning in data-rich but signal-ambiguous environments. AI is particularly relevant because it enables the integration of heterogeneous datasets, allowing simultaneous analysis of multiple physicochemical, operational, and sensor-derived variables, thereby improving anomaly detection robustness compared with conventional single-parameter monitoring approaches (5, 42, 43). A fundamental limitation of AI-based detection lies in the mismatch between model input signals and contamination specificity, where proxy variables do not uniquely encode wastewater intrusion, leading to structural uncertainty in model outputs (6). For example, ML models have demonstrated the ability to classify fecal contamination risk levels using combined fluorescence and physicochemical inputs, thereby reducing reliance on delayed laboratory confirmation (18). However, the value of AI in this domain remains conditional because wastewater-related signals are often subtle, transient, and embedded within high background variability. Model performance is highly dependent on data quality, representativeness, and validation design. Consequently, robust temporal validation and operational testing are essential before deployment in real-world systems. In drinking water systems, where contamination events are rare and datasets are often imbalanced, models may exhibit high apparent accuracy while failing under real-world conditions. This reinforces the need to position AI as an augmentation tool within a broader surveillance and decision-making framework rather than as a standalone solution. This positioning is essential to avoid over-reliance on predictive outputs without confirmatory validation and risk-based response mechanisms. AI-based approaches should be viewed as complementary rather than substitutive to conventional water testing techniques. Conventional laboratory methods provide high analytical specificity and regulatory confirmation but are constrained by sampling frequency and reporting delays. In contrast, AI-enabled systems offer rapid screening, anomaly detection, and rapid detection and response capabilities, but remain dependent on proxy indicators, sensor reliability, and validation quality. Consequently, effective contamination management is best achieved through integration of AI-assisted surveillance with confirmatory laboratory testing and established water-quality monitoring frameworks.

### 5.2 Machine learning approaches

ML methods form the core of AI-based applications in water quality assessment. These approaches are generally categorized into supervised and unsupervised learning, each addressing different aspects of contamination detection. Supervised learning models such as Random Forest (RF), Support Vector Machine (SVM), Extreme Gradient Boosting (XGBoost), and Artificial Neural Networks (ANN) are widely used for classification and regression tasks. These models require labeled datasets, where contamination events or water quality states are known. RF and gradient boosting models (e.g., XGBoost) are widely applied for anomaly detection and contamination classification using physicochemical and operational variables, particularly under structured or benchmark datasets, with reported performance ranges of *R*^2^ = 0.65–0.90 under controlled conditions. However, such performance often declines under real-world operational conditions due to variability in system behavior and reliance on non-specific proxy indicators (5, 6). SVM models are frequently applied for binary contamination classification tasks involving fecal-risk proxies and multivariate sensor signals, with reported accuracy typically ranging between 70 and 90% depending on dataset characteristics, feature selection, and validation design (19). Unsupervised learning approaches, including clustering algorithms and anomaly detection techniques, are applied when labeled data are unavailable. These methods identify deviations from normal system behavior, which may indicate potential contamination events. In drinking water systems, anomaly detection is especially important because confirmed contamination data are scarce. However, unsupervised models often generate false positives, particularly in systems with high natural variability, which can lead to alert fatigue in operational settings (5). Ensemble methods combine multiple models to improve predictive performance and robustness. For instance, hybrid frameworks integrating RF and ANN have been used to enhance contamination prediction accuracy by leveraging complementary strengths of different algorithms. This proxy dependence also affects model interpretability and reliability in operational settings. This limitation is particularly critical in operational settings, where incorrect classification may lead to either unnecessary interventions or missed contamination events. Reported AI performance metrics should therefore be interpreted within the context of contamination type, dataset characteristics, validation strategy, and operational deployment conditions. Classical ML models such as RF and XGBoost generally demonstrate strong performance for anomaly detection and physicochemical signal classification under structured datasets, whereas DL approaches such as LSTM are more effective for continuous temporal forecasting and multivariate time-series interpretation. However, many reported performance values are derived from benchmark datasets, simulation environments, or random train-test partitioning strategies that may not adequately represent the hydraulic variability and transient contamination dynamics of real drinking water systems. Furthermore, only a limited number of studies incorporate external validation, sustained field deployment, or evaluation under low-frequency contamination conditions. Consequently, predictive accuracy alone should not be interpreted as evidence of operational readiness without complementary temporal validation, confirmatory testing integration, and assessment of real-world deployment reliability.

### 5.3 Deep learning approaches

DL methods have gained attention for their ability to model complex temporal and nonlinear relationships in environmental datasets. Architectures such as Deep Neural Networks (DNN), Convolutional Neural Networks (CNN), and Long Short-Term Memory (LSTM) networks are increasingly applied to water quality time-series data. DL approaches, particularly LSTM networks, are primarily applied to continuous time-series forecasting and multivariate contamination trend analysis and have demonstrated high predictive performance in controlled datasets. However, their operational applicability remains constrained by substantial data requirements, limited interpretability, and reduced transferability across heterogeneous drinking water systems (20). To facilitate analytical comparison across model families, Table 4 summarizes representative AI approaches according to contamination indicators, validation strategies, operational applicability, evidence readiness, and major translational limitations.

**Table 4:** Comparative evaluation of representative AI models used for wastewater contamination monitoring in drinking water systems, including contamination indicators, validation strategies, operational applicability, evidence readiness, and translational limitations.

AI model	Primary application	Typical contamination indicator	Typical input data	Reported performance range	Validation strategy	Evidence readiness	Operational applicability	Key limitation(s)
RF	Contamination classification and anomaly detection	Turbidity, conductivity anomalies	Physicochemical, sensor, and operational variables	*R*^2^ = 0.65–0.90 under controlled datasets	Cross-validation; limited temporal validation	Experimental	Moderate	Performance declines under operational variability; proxy dependence
SVM	Binary contamination detection	Fecal contamination proxies	Multivariate sensor and laboratory data	Accuracy typically 70–90%	Cross-validation	Experimental	Moderate	Limited scalability and weak interpretability
LSTM	Time-series contamination prediction	Continuous multivariate fluctuations	Continuous sensor streams	*R*^2^ > 0.80 reported in benchmark datasets	Benchmark and simulation datasets; limited field validation	Prototype/pilot	Moderate–High	Data-intensive; limited transferability across systems
ANN	Water quality prediction and classification	Physicochemical variation patterns	Mixed environmental datasets	*R*^2^ = 0.60–0.85	Internal validation	Experimental	Moderate	Overfitting risk and black-box behavior
Hybrid ML + sensor systems	Real-time contamination risk assessment	Fluorescence-based organic signatures	Fluorescence and physicochemical sensors	80–85% classification accuracy in field-tested cases	Partial field validation	Field-tested	High	Calibration drift, sensor fouling, and site-specific dependence

Compiled and synthesized by the authors from the reviewed literature.

Overall, model selection should be guided not only by predictive performance but also by deployment maturity, interpretability, infrastructure requirements, and operational objectives. The comparative evaluation indicates that AI performance in water contamination assessment is strongly influenced by contamination characteristics, dataset composition, validation methodology, and operational context. Classical ML algorithms, including RF and Support Vector Machine (SVM), generally exhibit reliable performance for structured physicochemical datasets and anomaly classification tasks, while DL architectures are more effective for temporal forecasting and complex multivariate signal interpretation. Nevertheless, a large proportion of reported performance metrics are derived from controlled environments, benchmark datasets, or simulation-based studies with limited external validation. As a result, high predictive accuracy should not be considered sufficient evidence of operational readiness without long-term field evaluation, temporal robustness analysis, and integration with confirmatory monitoring frameworks.

Classical ML approaches provide advantages in computational efficiency, stability, and partial interpretability; however, their applicability in real-world systems is often constrained by dependence on non-specific proxy indicators and limited adaptability to dynamic operational conditions. In contrast, DL models such as LSTM networks demonstrate strong capability in capturing nonlinear temporal relationships and complex environmental interactions. CNNs, initially developed for image processing, have also been adapted for multivariate environmental data analysis, enabling automated hierarchical feature extraction and reducing the need for manual feature engineering (21). DL techniques further offer improved handling of missing observations and irregular sampling patterns, which are common challenges in water quality monitoring systems. Despite these advantages, several limitations restrict the broader operational adoption of DL frameworks. These models typically require extensive, high-quality datasets that are often unavailable in drinking water systems, particularly in resource-constrained settings. Their black-box structure also limits interpretability, reducing stakeholder confidence and complicating implementation in safety-critical applications. In addition, model generalization remains a major concern, as algorithms trained under one set of environmental and infrastructural conditions frequently exhibit reduced performance when transferred to other systems with different water chemistry, operational practices, or distribution characteristics. Hybrid sensing–AI frameworks appear to offer the greatest potential for real-time deployment by combining continuous sensor data acquisition with predictive analytics; however, their effectiveness remains dependent on sensor reliability, calibration stability, and site-specific optimization. Collectively, these findings emphasize that AI model selection should be guided not only by predictive performance, but also by data availability, interpretability, infrastructure compatibility, and operational objectives.

### 5.4 Hybrid and intelligent frameworks

Hybrid frameworks that integrate multiple data sources and modeling approaches represent an emerging direction in AI-based water quality assessment. These systems often combine sensor data, laboratory measurements, and operational parameters to improve detection accuracy and reliability. AI integrated with Internet of Things platforms enables continuous data collection and real-time analysis. Such systems can support rapid detection by identifying anomalies as they occur and triggering alerts for further investigation. In some cases, ML models are combined with fluorescence spectroscopy to enhance the detection of wastewater-derived organic matter, providing a more specific signal of contamination (11). Human-in-the-loop systems are also gaining importance. In these frameworks, AI-generated alerts are reviewed and validated by operators before action is taken. This approach balances automation with expert judgment, reducing the risk of false alarms while maintaining responsiveness. Physics-informed ML, although still in early stages, aims to incorporate domain knowledge into data-driven models. By embedding constraints related to hydraulic behavior or contaminant transport, these models can improve interpretability and reduce overfitting. However, their application in drinking water contamination assessment remains limited and requires further development.

### 5.5 Application domains

AI-based approaches in drinking water systems can be organized into distinct application domains based on the tasks they perform. It is important to distinguish between contamination, anomaly detection, prediction, and decision support because these terms represent different stages within the monitoring and response workflow. Contamination refers to the actual presence or intrusion of wastewater-derived hazards within a drinking water system. Anomaly detection identifies unusual patterns or deviations from expected system behavior that may indicate a potential contamination event but do not constitute confirmation. Prediction models estimate future contamination risk or system vulnerability based on historical and real-time data. Decision-support systems operate at a higher level by integrating monitoring outputs, predictive insights, confirmatory testing results, and operational objectives to guide response actions. Maintaining these distinctions is essential to avoid misinterpreting AI-generated alerts as direct evidence of contamination. Anomaly detection is one of the most common applications, where models identify deviations from baseline conditions that may indicate contamination. This is particularly useful for detecting sudden changes associated with intrusion events. Contamination classification involves distinguishing between different types of water quality anomalies, including those caused by wastewater intrusion. This requires models to learn specific signatures associated with wastewater-derived contaminants, which remains a challenge due to overlapping signals with other sources. Predictive modeling focuses on forecasting contamination risk based on historical data and system conditions. Such models can support proactive management by identifying high-risk periods or locations within the system. Decision support systems integrate AI outputs with operational workflows, enabling utilities to prioritize sampling, optimize response actions, and allocate resources more effectively. These systems are essential for translating model predictions into actionable interventions. Despite these applications, a recurring limitation is the lack of integration between AI outputs and confirmatory testing protocols. Without such integration, AI-based predictions may not translate into effective public health protection. Recent studies further demonstrate the practical potential of AI-enabled monitoring systems under applied conditions. Bedell et al. (9) reported successful near real-time classification of fecal contamination risk through integration of fluorescence sensing and machine-learning models in operational drinking water settings. Li et al. (5) highlighted the growing use of machine learning for anomaly detection and predictive management within drinking water distribution systems, particularly for identifying hydraulic disturbances and contamination-related signals. More recently, Aslan et al. (18) demonstrated the application of portable sensor-assisted analytical frameworks for rapid assessment of fecal pollution indicators, illustrating the increasing convergence of field-deployable sensing technologies and AI-supported decision-making. Collectively, these studies indicate that AI applications are gradually progressing beyond proof-of-concept research toward more operationally relevant monitoring scenarios, although widespread deployment remains limited.

### 5.6 Performance metrics and model evaluation

**Figure 5:**
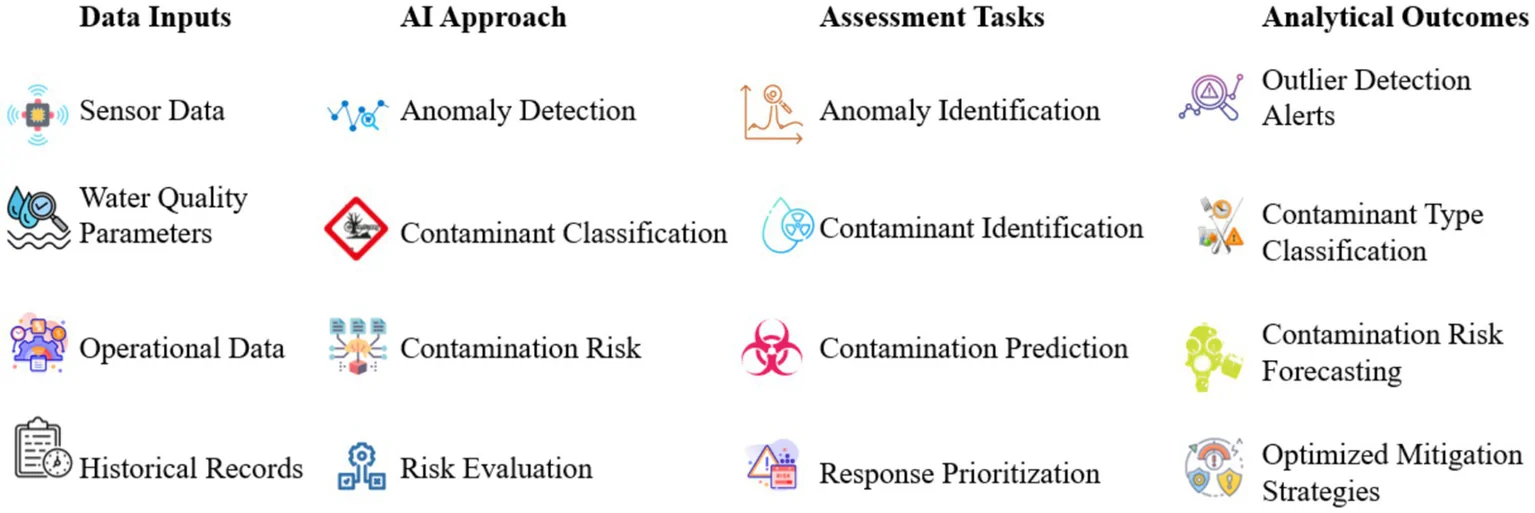
Comparative analytical framework linking AI model families with contamination detection tasks, input data structures, validation constraints, and operational applicability in drinking water systems. Developed by the authors based on the reviewed literature.

Model evaluation in AI-based water quality studies often relies on metrics such as coefficient of determination (R^2^), root mean square error (RMSE), mean absolute error (MAE), and classification metrics including accuracy, precision, recall, and F1-score. While these metrics provide quantitative measures of model performance, they are insufficient for assessing real-world applicability. Future evaluation frameworks must prioritize operational metrics such as detection latency, false alarm burden, and exposure reduction potential rather than relying solely on statistical accuracy measures (3). A critical limitation is the use of random train-test splits, which can inflate performance by ignoring temporal dependencies in the data. In drinking water systems, where conditions change over time, models must be evaluated using temporal validation strategies that reflect real operational scenarios. Class imbalance is another major challenge in AI-based environmental monitoring. Contamination events are typically rare relative to normal operating conditions, resulting in datasets heavily dominated by non-event observations. Consequently, models may achieve high overall accuracy while performing poorly in detecting the minority class representing actual contamination events. This issue is further compounded by data scarcity, as confirmed wastewater intrusion events with comprehensive sensor, operational, and laboratory records are rarely available for model training and validation. To address these challenges, future studies should incorporate balanced evaluation metrics such as precision, recall, F1-score, and area under the precision–recall curve, while exploring approaches such as data augmentation, anomaly detection, transfer learning, and semi-supervised learning to improve model robustness under limited-data conditions. Uncertainty quantification is often neglected in AI studies, despite its importance for decision-making. Models should provide confidence estimates alongside predictions to support risk-based actions. Additionally, robustness to concept drift, where system behavior changes over time, is rarely addressed but is essential for long-term deployment. False alarm burden is a key operational metric that is frequently overlooked. High false positive rates can reduce trust in AI systems and lead to alert fatigue, undermining their usefulness in practice. In addition, frequent false alarms can impose significant operational costs by triggering unnecessary sampling, system flushing, or emergency responses. These findings indicate that comparative evaluation of AI models must extend beyond statistical performance metrics to include contamination specificity, temporal robustness, deployment maturity, and public health relevance. Taken together, these limitations indicate that AI-enabled contamination monitoring should be interpreted as a decision-support augmentation framework rather than a fully autonomous replacement for conventional verification and public health response systems. Figure 5 illustrates how different AI methods align with specific contamination detection tasks and data types.

Figure 5 illustrates the mapping between AI model types, input data modalities, and specific contamination assessment tasks, highlighting the strengths and limitations of each approach. Following this conceptual mapping, Table 5 provides a detailed comparison of AI model families, including their inputs, outputs, validation strategies, and deployment maturity. The comparative evidence appraisal further distinguishes between proof-of-concept predictive studies and systems demonstrating partial or sustained operational deployment readiness.

**Table 5:** Evidence appraisal of representative AI-enabled wastewater contamination monitoring studies, highlighting contamination focus, validation design, operational strengths, translational limitations, and deployment readiness.

Study	System context	Contamination focus	Input data	AI approach	Analytical task	Output target	Validation strategy	Main operational strength	Major translational limitation	Evidence readiness
Ghobadi and Kang (8)	Water systems	Water quality deterioration	Physicochemical and historical datasets	RF, SVM	Prediction	Water quality index	Cross-validation	Robust predictive capability	Limited interpretability and weak external validation	Experimental
Li et al. (5)	Distribution systems	Distribution-system anomaly signals	Sensor and operational data	XGBoost	Anomaly detection	Contamination signal identification	Temporal split validation	High anomaly-detection sensitivity	Proxy-variable dependence and site specificity	Pilot
Bedell et al. (9)	Drinking water systems	Fecal contamination risk	Fluorescence and sensor data	ML classifier	Risk classification	Fecal contamination probability	Field validation	Near real-time detection capability	Calibration sensitivity and maintenance needs	Field-tested
Dharmarathne et al. (11)	IoT-based monitoring systems	Real-time water quality anomalies	Continuous sensor streams	ANN	Monitoring and prediction	Water quality status	Simulation + limited field testing	continuous monitoring integration potential	Data reliability and transferability constraints	Prototype
Zhi et al. (21)	Environmental monitoring systems	Temporal contamination dynamics	Multivariate environmental data	LSTM, CNN	Prediction	Temporal contamination trends	Benchmark datasets	Strong nonlinear temporal modeling	Data-intensive architecture and limited operational deployment	Experimental

Compiled and synthesized by the authors from the reviewed literature.

The evidence appraisal indicates that the majority of AI-driven wastewater contamination studies remain confined to experimental setups, simulation-based analyses, or short-term pilot investigations. Only a limited number of studies incorporate long-term field validation, cross-system external testing, or direct integration with operational public health response frameworks. This imbalance highlights that the current evidence base is considerably more developed for predictive experimentation than for demonstrating sustained deployment readiness in practical drinking water systems. The comparison further shows that reported AI performance varies substantially according to contamination type, dataset composition, validation methodology, and operational context. While numerous studies achieve high predictive accuracy under controlled or benchmark conditions, relatively few evaluate long-term reliability, external transferability, or continuous real-world applicability. Consequently, the existing literature provides stronger support for proof-of-concept predictive performance than for robust operational implementation. Moreover, despite the promising predictive capabilities demonstrated by AI models, their technological and deployment maturity remains limited. Most studies are still conducted in controlled or semi-controlled environments, with scarce evidence of continuous field-scale operation. Bridging the gap between experimental success and practical deployment therefore represents a major challenge for the field. Addressing this limitation will require rigorous validation protocols, improved integration with real-time monitoring and decision-support systems, and closer alignment with public health and regulatory objectives. Building on this perspective, the following section examines the integration of AI with smart sensing technologies to facilitate real-time surveillance and early warning in drinking water systems.

## 6 Smart sensing, online monitoring, and AI-enabled water surveillance systems

### 6.1 Sensor-based monitoring

The transition from periodic sampling to continuous monitoring has been a critical shift in drinking water quality assessment. Sensor-based monitoring systems enable high-frequency data acquisition, allowing detection of rapid changes associated with wastewater contamination events. These systems typically rely on surrogate parameters such as turbidity, conductivity, pH, oxidation–reduction potential, and residual chlorine, which can be measured in real time using *in situ* probes. Among advanced sensing approaches, fluorescence spectroscopy has emerged as a promising tool for detecting wastewater-derived organic matter. Fluorescent dissolved organic matter exhibits characteristic excitation–emission signatures that can act as proxies for fecal contamination. Field-deployable fluorescence sensors have demonstrated the ability to capture dynamic changes in water quality, providing near real-time insights into contamination processes (9). Electrochemical and optical sensors further expand monitoring capabilities by enabling detection of specific chemical and biological signals. Biosensors, although still under development, offer potential for direct detection of microbial contaminants or metabolic activity. However, their application in drinking water systems remains limited due to issues related to stability, sensitivity, and long-term deployment. Recent technological developments indicate increasing commercialization of smart water-quality monitoring platforms. For example, open-source systems such as WaterScope have been developed to facilitate rapid microbial water-quality assessment under resource-constrained conditions and have demonstrated potential for field deployment (22). Similarly, IoT-enabled multi-parameter monitoring platforms integrating turbidity, conductivity, pH, and residual chlorine sensors are increasingly being evaluated in pilot-scale drinking water surveillance systems for continuous anomaly detection and remote monitoring. Although large-scale operational adoption remains limited, these examples illustrate the growing transition from laboratory-based sensing concepts toward deployable monitoring technologies. Despite these advances, sensor-based monitoring is constrained by practical challenges.

Sensor-based monitoring is limited by fouling, calibration drift, and environmental interference, which can degrade measurement accuracy and propagate uncertainty into AI-based inference under fluctuating baseline conditions. These limitations highlight that sensor data must be continuously validated and corrected to maintain reliability.

### 6.2 AI integration with real-time systems

The integration of AI with sensor networks has enabled the development of continuous water surveillance systems capable of early warning and decision support. These systems operate through a multi-stage pipeline that includes data acquisition, preprocessing, model inference, alert generation, and response activation. In practice, raw sensor data are first subjected to quality control procedures to remove noise and outliers. ML models are then applied to detect anomalies or classify contamination risk based on learned patterns. Alerts generated by these models are typically categorized into different severity levels, allowing operators to prioritize responses. A critical component of such systems is the linkage between AI outputs and confirmatory testing. Detection alone is insufficient; it must be followed by targeted sampling and laboratory analysis to verify contamination and guide intervention. This aligns with contamination warning system frameworks, which emphasize the integration of monitoring, detection, confirmation, and response (3). Real-time AI systems also support predictive capabilities, enabling utilities to anticipate contamination risks based on system conditions. For example, models can incorporate hydraulic and operational data to identify periods of increased vulnerability, such as low-pressure events or maintenance activities. However, the reliability of such predictions depends on the availability of high-quality, system-specific data.

### 6.3 Opportunities for LMIC settings

LMIC face significant challenges in maintaining safe drinking water systems due to limited infrastructure, intermittent supply, and constrained monitoring capacity. In these contexts, AI-enabled sensing systems offer opportunities to enhance surveillance while reducing reliance on resource-intensive laboratory methods. Low-cost sensor platforms combined with ML models can provide decentralized monitoring solutions that are scalable and adaptable to local conditions. Open-source technologies and portable devices have been developed to enable rapid assessment of water quality, including microbial contamination, with minimal technical requirements (22). Examples such as the WaterScope platform demonstrate how portable and relatively low-cost microbial monitoring systems can support decentralized surveillance in low-resource settings, potentially complementing AI-assisted interpretation and risk prioritization. Such systems can improve early detection of contamination events, particularly in informal settlements where conventional monitoring is often absent. By providing real-time data, they support timely interventions and reduce exposure risks. Additionally, digital integration allows for centralized data aggregation and analysis, facilitating broader surveillance and decision-making. However, the deployment of these technologies must consider local constraints, including maintenance capacity, availability of spare parts, and user training. Economic constraints, including the costs of sensor procurement, calibration, maintenance, and digital infrastructure, may further limit large-scale adoption. In addition, regulatory barriers such as the absence of standardized validation protocols, limited data-governance frameworks, and lack of guidance for AI-assisted decision-making can hinder integration into routine drinking water monitoring programs. Addressing these challenges will require coordinated investment in infrastructure, technical capacity building, and regulatory support mechanisms. In LMIC contexts, intermittent power supply, limited sensor maintenance capacity, and frequent data gaps further constrain the sustainability and reliability of AI-based monitoring systems.

### 6.4 Limitations

**Figure 6:**
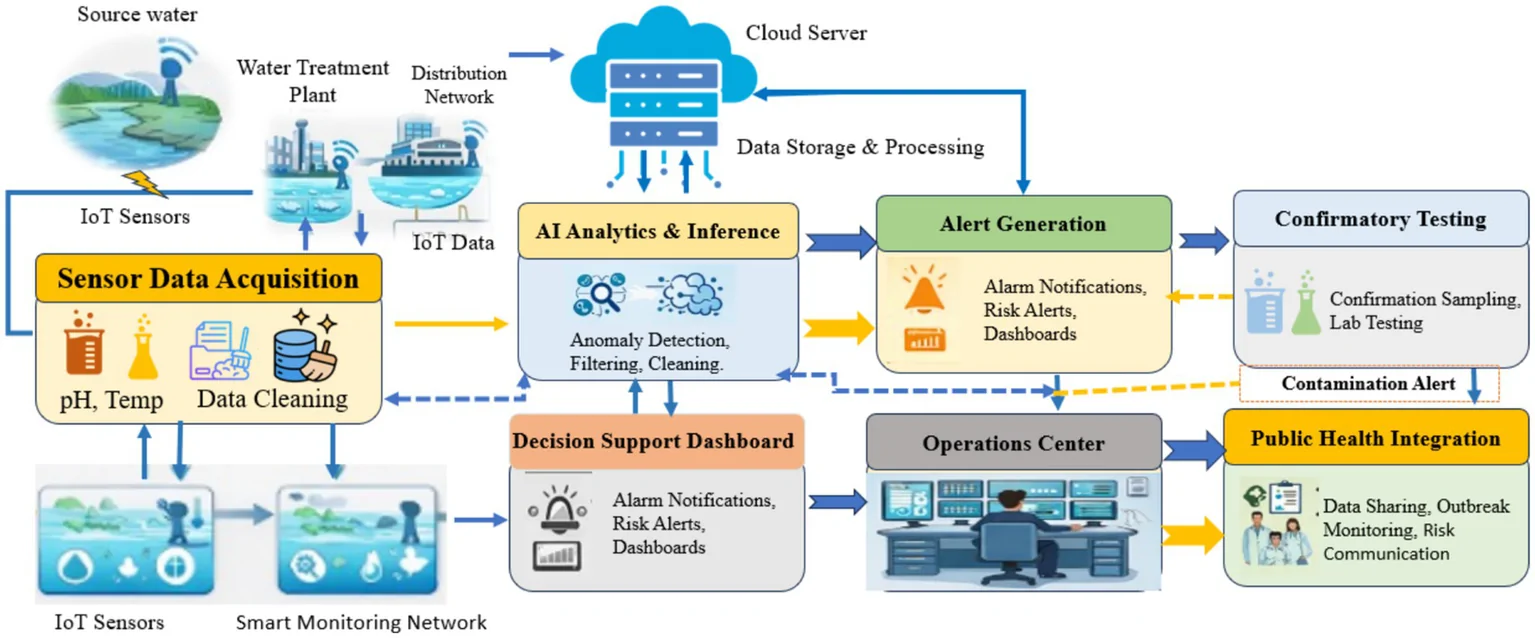
End-to-end AI-enabled surveillance architecture illustrating sensor integration, data preprocessing, predictive analytics, anomaly detection, confirmatory testing, and operational response workflows in drinking water systems. Developed by the authors based on the reviewed literature.

While AI-enabled water surveillance systems offer significant potential, their practical implementation is constrained by several factors. Because sensor fouling, drift, and environmental interference were discussed earlier, this section focuses on their operational implications, including maintenance burden, data continuity, and long-term deployment reliability. Data continuity is another critical issue. Interruptions in data streams due to power outages, network failures, or equipment malfunction can compromise model predictions and reduce system effectiveness. In many settings, especially in LMICs, such disruptions are frequent and difficult to manage. Cybersecurity and data governance also present emerging challenges. As water monitoring systems become increasingly digitized, they become vulnerable to cyber threats that can disrupt operations or manipulate data. Ensuring secure data transmission and storage is essential for maintaining trust in AI-based systems. Operational integration poses additional challenges. AI-generated alerts must be interpreted and acted upon by trained personnel. Without clear protocols and sufficient capacity, the benefits of early warning may not translate into effective response. Over-reliance on automated alerts without human validation can further amplify this risk, particularly in systems with high baseline variability. Alert fatigue, caused by frequent false positives, can further reduce the responsiveness of operators. Figure 6 illustrates the architecture of AI-enabled water surveillance systems, highlighting the flow from sensing to decision-making and response.

Figure 6 illustrates the end-to-end pipeline of AI-enabled surveillance, including sensor data acquisition, preprocessing, model inference, alert generation, confirmatory testing, and operational response. Following this conceptual framework, Table 6 provides a detailed comparison of sensing modalities, their operational benefits, and associated failure modes in real-world deployment.

**Table 6:** Smart sensing modalities, operational benefits, and real-world failure modes in AI-enabled drinking water surveillance.

Sensor and modality	Target parameters	Typical use	AI integration potential	Strength	Maintenance issue	Failure mode	Suitability for low-resource settings	References
Turbidity sensor	Suspended solids	Continuous monitoring	High	Real-time detection	Fouling	Drift	High	(5)
Conductivity probe	Ionic changes	Mixing detection	High	Sensitive to intrusion	Calibration drift	Noise	High	(5)
Fluorescence sensor	Organic matter	Wastewater proxy	High	Specific signal patterns	Fouling	Signal interference	Moderate	(9)
Electrochemical sensor	Chemical species	Targeted detection	Moderate	High sensitivity	Electrode degradation	Instability	Moderate	(11)
Biosensor	Microbial activity	Pathogen detection	Emerging	Direct detection	Limited durability	Low reproducibility	Low	(22)
IoT sensor network	Multi-parameter	Integrated monitoring	High	Scalable systems	Connectivity issues	Data gaps	Moderate	(11)

Compiled and synthesized by the authors from the reviewed literature.

The table highlights that while sensor technologies provide critical inputs for AI-based systems, their effectiveness is closely tied to operational reliability and maintenance capacity. No single sensing modality can capture all aspects of wastewater contamination, necessitating the use of multi-parameter monitoring frameworks. Importantly, the integration of sensing and AI must be guided by system-specific considerations, including infrastructure characteristics, resource availability, and governance capacity. Without such alignment, the potential benefits of real-time surveillance may not be realized in practice. This section establishes that smart sensing and AI integration are essential components of modern water surveillance systems, but their success depends on addressing practical and operational challenges. The next section builds on this foundation by examining how wastewater contamination translates into public health risks and how AI can support risk mitigation.

## 7 Public health implications of wastewater contamination in drinking water systems

### 7.1 Exposure pathways and vulnerable populations

Wastewater contamination in drinking water systems represents a direct pathway for human exposure to a complex mixture of microbial and chemical hazards. The dominant exposure route is ingestion through drinking water, but additional pathways include food preparation, oral hygiene, and dermal contact during domestic use. In certain conditions, aerosolization during household activities such as showering may also contribute to exposure, particularly for opportunistic pathogens present in premise plumbing systems. Exposure risk is not uniformly distributed across populations. Vulnerable groups include children, older adults, and immunocompromised individuals, who are more susceptible to infection and adverse health outcomes. Communities in low- and middle-income settings face additional risks due to intermittent water supply, inadequate sanitation infrastructure, and limited monitoring capacity. These factors increase the likelihood of contamination events and prolong exposure duration. Evidence from global assessments indicates that fecal contamination remains a widespread issue in drinking water supplies, particularly in regions with inadequate water safety management systems. According to the World Health Organization, contamination of drinking water with fecal matter continues to contribute significantly to the global burden of diarrheal diseases, with children under 5 years of age being disproportionately affected (2, 23).

### 7.2 Microbial health risks

Microbial contamination associated with wastewater intrusion poses immediate and acute health risks. Infectious doses for several enteric pathogens can be as low as 10–100 organisms, meaning that even short-duration contamination events can result in significant health risk if not detected promptly (1). Fecal indicator bacteria such as *Escherichia coli* serve as proxies for the presence of pathogenic microorganisms, including viruses, protozoa, and bacteria capable of causing gastrointestinal illness. Outbreak investigations have demonstrated that failures in drinking water systems, including distribution system breaches and cross-connections, can lead to widespread disease transmission. Surveillance data from multiple countries have identified drinking water as a recurrent source of outbreaks, particularly when contamination is not detected promptly (7). Opportunistic pathogens present an additional concern, especially in premise plumbing systems. Organisms such as Legionella and Pseudomonas can proliferate within biofilms and may not be directly associated with fecal contamination. Wastewater intrusion can alter microbial community dynamics, facilitate the growth of such pathogens and increasing infection risk. Recent studies have shown that even short-term exposure to wastewater-contaminated water can lead to significant shifts in microbial composition, enhancing the presence of pathogenic and antibiotic-resistant organisms. These findings highlight the need for monitoring approaches that capture not only indicator organisms but also broader microbial dynamics (24).

### 7.3 Chemical and chronic health risks

In addition to microbial hazards, wastewater contamination introduces a range of chemical contaminants into drinking water systems. These include pharmaceuticals, personal care products, endocrine-disrupting compounds, and industrial chemicals. While concentrations are often low, chronic exposure to such contaminants raises concerns about long-term health effects. Wastewater-derived organic matter can also influence the formation of disinfection by-products during water treatment and distribution. Changes in organic composition may increase the formation potential of compounds such as trihalomethanes and haloacetic acids, which are associated with carcinogenic and reproductive health risks. Heavy metals and inorganic contaminants may also be introduced through wastewater pathways, particularly in systems affected by industrial discharge. Although less common than microbial contamination, these contaminants can contribute to cumulative exposure and chronic health impacts. A key challenge in assessing chemical risks is the presence of complex mixtures. Traditional risk assessment approaches often consider individual contaminants, whereas wastewater contamination involves multiple interacting substances. This complexity complicates both detection and health impact evaluation, necessitating more integrated assessment frameworks.

### 7.4 Broader public health burden

The public health implications of wastewater contamination extend beyond individual health outcomes to include broader societal impacts. Delayed detection of contamination events can lead to large-scale outbreaks, placing significant strain on healthcare systems. In addition, repeated incidents undermine public trust in drinking water systems, which can result in behavioral changes such as increased reliance on bottled water or alternative sources. Inequities in water quality monitoring and infrastructure further exacerbate these impacts. Populations with limited access to safe water are more likely to experience repeated exposure, contributing to persistent health disparities. In such contexts, contamination events may go undetected or unreported, leading to underestimation of the true disease burden. The economic implications are also significant. Costs associated with healthcare, emergency response, and system remediation can be substantial, particularly in large-scale contamination events. These factors highlight the importance of proactive monitoring and prevention strategies in reducing public health risks.

### 7.5 How AI can support public health protection

**Figure 7:**
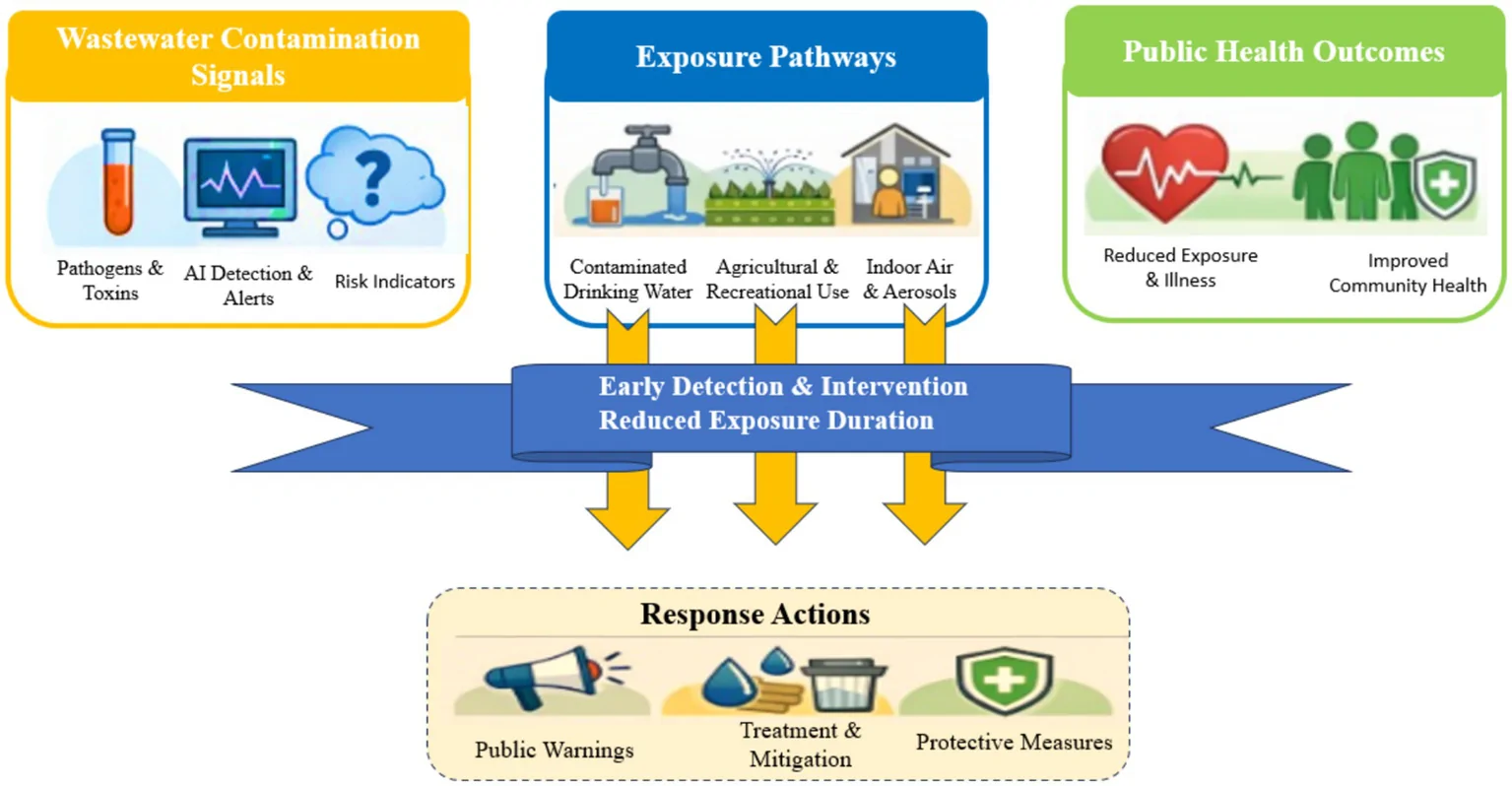
Translational framework linking contamination signals, exposure pathways, confirmatory interpretation, intervention strategies, and public health protection outcomes in drinking water systems. Developed by the authors based on the reviewed literature.

AI has the potential to enhance public health protection by improving the timeliness and accuracy of contamination detection. By analyzing continuous sensor data, AI models can identify early signals of contamination, enable faster response and reduce exposure duration. AI can also support prioritization of confirmatory testing by identifying high-risk events or locations within the system. AI outputs must be explicitly linked to predefined public health response thresholds, such as triggering boil water advisories, targeted microbial testing, or system flushing interventions. This targeted approach improves the efficiency of monitoring programs and ensures that resources are allocated where they are most needed. Integration of AI with public health surveillance systems can further enhance risk management. For example, linking water quality data with disease surveillance data may enable early identification of correlations between contamination events and health outcomes. Such integration supports a more holistic approach to water safety, aligning environmental monitoring with public health objectives. However, the effectiveness of AI in this context depends on its integration with governance frameworks. Models must be transparent, validated, and aligned with established protocols for confirmatory testing and response. Without such integration, AI outputs may not translate into actionable public health interventions. Figure 7 illustrates the relationship between contamination signals, exposure pathways, and health outcomes, highlighting where AI can intervene to reduce risk.

Figure 7 illustrates how contamination signals detected through monitoring systems translate into exposure pathways, health risks, and intervention points, with AI-enabled systems supporting early detection and response. Following this conceptual framework, Table 7 presents a structured interpretation of contamination signals in relation to exposure pathways, health risks, and response actions.

**Table 7:** Public health interpretation of wastewater contamination signals in drinking water systems: exposure pathways, health concerns, confirmatory needs, and response implications.

Signal type	Likely interpretation	Exposure pathway	Vulnerable groups	Health relevance	Confirmatory testing	Response implication	References
Elevated turbidity	Possible intrusion or disturbance	Ingestion	Children, older adults	Moderate	Microbial testing	System inspection	(39)
Low chlorine residual	Increased microbial risk	Ingestion	All populations	High	Culture/qPCR	Flushing, disinfection	(40)
Fluorescence signal shift	Wastewater-derived organics	Ingestion	General population	Moderate	Spectroscopy, lab analysis	Source identification	(41)
*E. coli* detection	Fecal contamination	Ingestion	High-risk groups	High	Culture confirmation	Boil water advisory	(2)
Pathogen DNA (qPCR)	Presence of specific pathogens	Ingestion	Immunocompromised	High	Molecular confirmation	Targeted intervention	(24)
Chemical anomaly	Possible industrial contamination	Ingestion	General population	Chronic risk	Chromatography	Source control	(6)

Compiled and synthesized by the authors from the reviewed literature.

The table demonstrates that effective public health protection requires not only detection of contamination signals but also their correct interpretation and linkage to appropriate response actions. Each signal must be evaluated within the context of exposure pathways and system conditions to determine its significance. This section establishes that wastewater contamination in drinking water systems poses multifaceted public health risks, encompassing both acute and chronic impacts. It also highlights the potential of AI to enhance surveillance and response, while emphasizing the need for integration with established public health frameworks. The next section critically evaluates the current AI literature, identifying strengths, limitations, and barriers to real-world implementation.

### 7.6 Translating AI outputs into public health action thresholds

**Figure 8:**
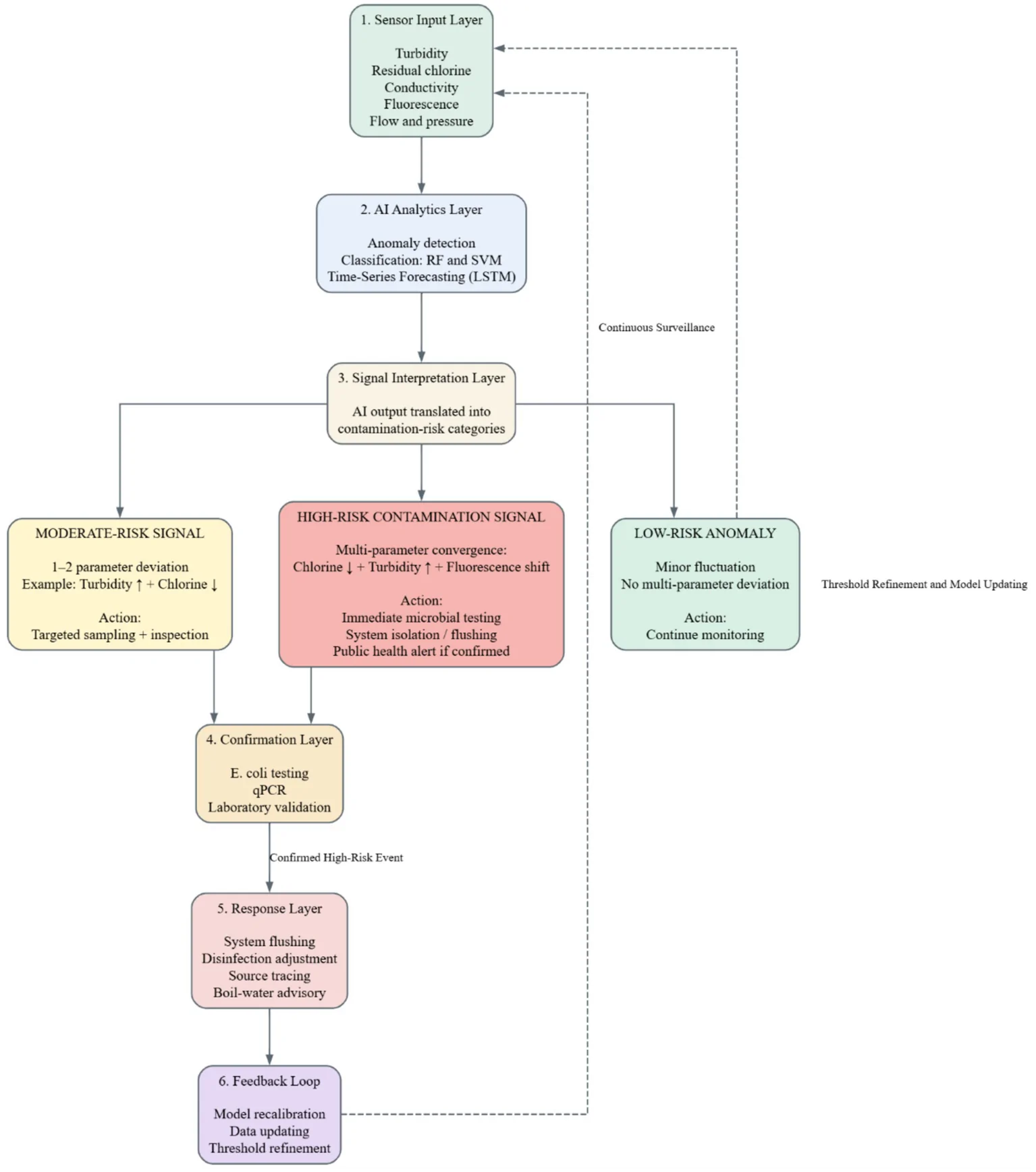
Decision-oriented AI framework linking anomaly detection, confirmatory testing, risk categorization, and tiered public health response actions in drinking water systems. Developed by the authors based on the reviewed literature.

A major limitation of current AI-based monitoring frameworks is the absence of a clear and operational linkage between model outputs and actionable public health responses. While AI enables detection of anomalies and prediction of contamination risk, these outputs often remain abstract and disconnected from decision-making processes within drinking water systems. For AI to be operationally meaningful, anomaly detection must be translated into predefined decision thresholds explicitly aligned with risk-based water safety planning and response protocols. In drinking water systems, contamination risk is rarely indicated by a single parameter but rather by the convergence of multiple signals. For instance, a simultaneous decrease in residual chlorine, increase in turbidity, and shift in fluorescence signatures represents a high-probability wastewater intrusion signal. Such multi-parameter convergence provides stronger diagnostic confidence than isolated indicators and should trigger tiered response actions. These actions include (i) immediate confirmatory microbial testing (e.g., *E. coli* or qPCR), (ii) targeted operational interventions such as system flushing, isolation, or disinfection adjustment, and (iii) escalation to public health advisories where contamination is confirmed or risk remains high. In contrast, lower-level anomalies involving single-parameter deviations may warrant enhanced monitoring or targeted inspection rather than immediate system-wide intervention. This tiered response structure reflects a necessary shift from detection-centric AI applications toward decision-oriented frameworks that integrate detection, confirmation, and response within a unified workflow. Embedding AI outputs within structured response protocols reduces ambiguity in interpretation, limits false alarm burden, and ensures that early warning signals translate into timely and proportionate interventions. Such integration is critical for minimizing exposure duration and strengthening the protective function of drinking water monitoring systems. To operationalize this concept, a structured AI-driven decision framework is presented in Figure 8.

Figure 8 illustrates a unified workflow connecting sensor-based monitoring, AI-driven anomaly detection, signal interpretation, confirmatory testing, and response actions within a single system. Multi-parameter anomaly patterns are classified into risk categories and mapped to predefined intervention thresholds, enabling tiered decision-making. The framework also incorporates a feedback loop for continuous model recalibration and threshold refinement, ensuring adaptability under changing system conditions. By explicitly linking AI outputs to response pathways, the framework addresses a critical translational gap and provides a practical foundation for integrating AI into water safety planning and public health protection. AI-enabled monitoring systems provide potential public health benefits not only through earlier contamination detection but also through improved operational decision support. Real-time anomaly detection can reduce detection latency and support rapid confirmatory testing before widespread exposure occurs. Similarly, integration of AI-generated alerts with hydraulic and distribution-system data may enable targeted isolation of vulnerable network zones, reducing exposure duration during intrusion events. AI-assisted prioritization of sampling locations may further improve resource allocation and strengthen boil-water advisory decisions during suspected contamination episodes. However, these benefits remain dependent on temporal validation, low false-alarm rates, and integration with established public health response protocols. Consequently, the practical value of AI lies not solely in predictive accuracy but in its ability to support actionable risk-reduction pathways within operational drinking water management systems (Table 8).

**Table 8:** Evidence-readiness classification framework summarizing methodological maturity, operational validation status, and translational limitations of AI-based wastewater contamination monitoring studies.

Evidence category	Typical study type	Validation level	Operational relevance	Major limitation
Simulation and benchmark studies	Public datasets, synthetic contamination	Cross-validation only	Low	Limited real-world realism
Laboratory studies	Controlled contamination experiments	Internal validation	Moderate	Site-independent uncertainty
Pilot field studies	Sensor-integrated monitoring	Partial temporal validation	Moderate–High	Short deployment duration
Operationally integrated systems	Utility-linked workflows	Real-world deployment	High	Rare in current literature

Compiled and synthesized by the authors from the reviewed literature.

## 8 Critical appraisal of current AI literature: strengths, gaps, and translational barriers

### 8.1 Major advances

Recent advances in AI applications for drinking water systems demonstrate a clear shift from static water quality assessment toward dynamic, data-driven surveillance. One of the most significant contributions is the ability of ML models to integrate heterogeneous datasets, including sensor signals, operational parameters, and environmental variables, into unified predictive frameworks. This integration enables detection of subtle multivariate patterns associated with contamination events that would otherwise remain undetected through conventional monitoring. Field-based studies have further strengthened the credibility of AI approaches by demonstrating near real-time classification of fecal contamination risk using combined sensor inputs. Such approaches move beyond theoretical modeling and provide evidence of operational feasibility in controlled environments. Additionally, advances in time-series modeling, particularly with recurrent neural networks, have improved the ability to capture temporal dependencies and forecast contamination trends under varying system conditions. Another important development is the emergence of hybrid frameworks that combine AI with sensing technologies and decision-support systems. These frameworks reflect a transition from isolated model development to integrated surveillance architectures, where AI supports detection, prioritization, and response. This evolution aligns with the broader objective of reducing time-to-detection and improving system resilience.

### 8.2 Key methodological limitations

Despite these advances, the current body of AI literature exhibits several critical systemic methodological deficiencies that limit translational reliability and real-world deployment. These limitations reduce its applicability in real-world drinking water systems. Across the reviewed literature, over 70% of studies rely on physicochemical proxy variables such as turbidity and conductivity, while fewer than 20% incorporate temporal validation or real-world deployment testing, indicating a substantial gap between model development and operational applicability (5, 11). One of the most pervasive issues is the reliance on proxy variables that lack specificity for wastewater contamination. This creates ambiguity in model outputs and increases the risk of misclassification. Some studies report strong predictive performance when using surrogate indicators such as turbidity, conductivity, dissolved organic carbon, or fluorescence-derived signals as model inputs. However, other studies have highlighted that these parameters are not uniquely associated with wastewater intrusion and may also respond to natural environmental variability, hydraulic disturbances, or operational changes within distribution systems. Consequently, while proxy-based approaches can improve anomaly detection sensitivity, disagreement remains regarding their contamination specificity and reliability for operational decision-making. This divergence in findings suggests that model performance is strongly influenced by local system characteristics, sensor configuration, and validation design, emphasizing the need for multi-signal confirmation strategies rather than reliance on individual proxy indicators alone. Parameters such as turbidity, conductivity, and organic carbon can indicate changes in water quality but do not uniquely represent contamination from wastewater sources. The operational implications of this limitation include increased uncertainty in contamination attribution and reduced decision confidence. In real-world systems, such misclassification can directly translate into either unnecessary operational actions or failure to detect actual contamination events. False-positive alerts may trigger unnecessary sampling, flushing, or other operational interventions, increase costs and contribute to alert fatigue among operators. Conversely, false-negative outcomes may delay contamination recognition, prolong exposure duration, and reduce the effectiveness of public health response measures. Consequently, proxy-based AI predictions should be interpreted alongside confirmatory monitoring and operational context rather than as standalone evidence of contamination events. Model validation practices also present significant concerns. Many studies employ random train–test splits, which do not account for temporal dependencies inherent in water quality data. This approach can lead to inflated performance metrics and does not reflect the conditions under which models would operate in practice. Temporal validation, where models are tested on future data relative to the training period, is rarely implemented but is essential for assessing predictive reliability. Class imbalance further complicates model development. Contamination events are relatively rare compared to normal operating conditions, resulting in datasets dominated by non-event observations. Models trained on such datasets may achieve high overall accuracy while failing to detect actual contamination events, which are the most critical cases. Uncertainty quantification is another area of concern. Many AI models provide point predictions without associated confidence intervals, limiting their usefulness for risk-based decision-making. In the context of public health, where decisions may have significant consequences, the absence of uncertainty information undermines trust and interpretability.

### 8.3 Data and infrastructure constraints

The effectiveness of AI models is fundamentally constrained by the availability and quality of data. In drinking water systems, labeled datasets representing confirmed contamination events are scarce. This scarcity limits the ability of supervised models to learn meaningful patterns and increases reliance on synthetic or simulated data, which may not accurately represent real-world conditions. Data heterogeneity presents additional challenges. Sensor data are often collected at different temporal resolutions, with varying levels of accuracy and completeness. Inconsistent metadata, including information on sensor calibration, maintenance, and environmental conditions, further complicates data integration and interpretation. Infrastructure limitations also play a role. Many water utilities lack the digital infrastructure required to support continuous data collection and analysis. Even where sensors are deployed, data management systems may be insufficient to handle large volumes of high-frequency data. This restricts the scalability of AI-based approaches and limits their adoption. Interoperability between systems remains a significant barrier. Data generated by different sensors and platforms are often incompatible, preventing seamless integration and analysis. Standardization of data formats and protocols is necessary to enable effective use of AI in water quality monitoring.

### 8.4 Real-world implementation barriers

Translating AI models from research to practice involves overcoming several operational challenges. One of the most prominent issues is alert fatigue, where frequent false positives reduce the responsiveness of operators. In systems with high variability, distinguishing between true contamination events and benign fluctuations is difficult, leading to either overreaction or complacency. Operator trust is a critical factor, and the lack of interpretability and transparency in model outputs remains a major barrier to adoption in safety-critical infrastructure systems. Without transparency and interpretability, adoption of AI-based systems may be limited, particularly in risk-sensitive applications such as drinking water safety. Cost and resource requirements also influence implementation. While AI models themselves may be computationally efficient, their deployment requires investment in sensors, data infrastructure, and skilled personnel. In many cases, these requirements exceed the capacity of smaller utilities or those in resource-constrained settings. Sustainability of deployment is an additional concern. Maintaining sensor networks, updating models, and ensuring data quality over time require ongoing effort and resources. Without long-term planning and support, initial deployments may fail to deliver sustained benefits.

### 8.5 Ethical and governance concerns

The integration of AI into drinking water surveillance systems raises important ethical and governance considerations. Transparency is essential to ensure that model outputs can be understood and audited. Explainable artificial intelligence (XAI) approaches can help address this challenge by providing interpretable reasoning behind model predictions. Techniques such as SHAP (Shapley Additive Explanations), LIME (Local Interpretable Model-Agnostic Explanations), and feature-importance analysis allow operators to identify the variables most strongly influencing contamination-risk predictions. Such approaches improve transparency, facilitate expert review, and strengthen stakeholder confidence in AI-assisted monitoring systems. From a regulatory perspective, explainability is particularly important because water-safety decisions often require documented justification and auditability. Consequently, AI systems are more likely to gain regulatory acceptance when deployed as decision-support tools operating under human oversight rather than as fully autonomous decision-making systems. This is particularly important in public health contexts, where decisions based on AI predictions may affect large populations. Algorithmic accountability is another key issue. Responsibility for decisions made based on AI outputs must be clearly defined, including the roles of model developers, system operators, and regulatory authorities. Without clear accountability, there is a risk of misinterpretation or misuse of model predictions. Data governance is equally important. Water quality data, particularly when integrated with public health information, must be managed in a way that ensures privacy, security, and integrity. Cybersecurity risks are increasing as water systems become more digitized, making it essential to protect data from unauthorized access or manipulation. Finally, communication of AI-generated insights must be handled carefully. Public health decisions require clear and accurate information, and miscommunication can lead to unnecessary panic or complacency. Establishing protocols for communicating risks and uncertainties is therefore essential for responsible use of AI in this domain. This critical appraisal highlights that while AI offers significant potential for improving wastewater contamination detection and management, its current application is limited by methodological weaknesses, data constraints, and implementation challenges. Addressing these issues is essential for translating research advances into practical solutions that enhance public health protection. The next section outlines future directions and research priorities aimed at overcoming these limitations and advancing the field toward more reliable and impactful applications.

### 8.6 Evidence readiness and translational reliability of AI studies

A recurring limitation across the reviewed literature is the mismatch between reported predictive performance and demonstrated operational readiness. Although many studies report high classification accuracy or strong predictive metrics under controlled conditions, comparatively few evaluate model robustness under real-world deployment scenarios. Most published studies remain concentrated within laboratory-scale experiments, benchmark datasets, simulation environments, or short-term pilot investigations. Only a limited number incorporate sustained field validation, temporal robustness assessment, or integration with operational decision workflows. A similar divergence is observed between experimental and operational studies. While simulation-based and laboratory investigations frequently report high predictive performance under controlled conditions, field deployments often identify additional challenges related to sensor maintenance, calibration drift, data gaps, and changing hydraulic conditions. These differences highlight that predictive success under controlled environments does not necessarily translate into equivalent operational performance in real-world drinking water systems. The reviewed literature can broadly be categorized into four evidence-readiness groups. The first group consists of simulation-based or benchmark-data studies that primarily evaluate algorithmic performance under controlled conditions. These studies contribute methodological development but provide limited evidence regarding operational applicability. The second group includes laboratory or semi-controlled experimental studies integrating sensors with ML models, often demonstrating proof-of-concept contamination detection under defined environmental conditions. The third group comprises short-term pilot or field-tested systems that evaluate AI-assisted monitoring under partially operational conditions, although many remain limited by site specificity and constrained validation duration. The fourth and least represented category includes operationally integrated systems linked with confirmatory testing, decision-support workflows, or utility-scale implementation. Across these categories, several recurring methodological weaknesses were identified. Temporal validation remains insufficiently implemented, with many studies relying on random train-test splits that may overestimate predictive reliability under dynamic operational conditions. External validation across independent systems is also rare, limiting model transferability and scalability. In addition, relatively few studies explicitly quantify uncertainty, evaluate false alarm burden, or assess long-term robustness against concept drift and changing hydraulic conditions. These findings indicate that current AI-based monitoring research remains substantially stronger in predictive experimentation than in demonstrated operational translation. Consequently, claims regarding real-world applicability should be interpreted cautiously unless supported by field validation, sustained deployment testing, and integration with structured public health or water safety response frameworks.

These translational gaps directly inform the future research priorities discussed in the following section.

## 9 Future perspectives

Future progress in AI-based assessment of wastewater contamination in drinking water systems must transition from model-centric development toward operationally integrated, decision-oriented frameworks. A key next-generation direction is the coupling of AI-driven monitoring with Digital Twin architectures, enabling real-time simulation of hydraulic dynamics, contaminant transport, and intervention outcomes under variable system conditions. Such integration can transform static monitoring into predictive, system-aware surveillance capable of anticipating contamination risks before exposure occurs. A central priority is the development of explainable and trustworthy AI systems. Future research should also evaluate how XAI frameworks can support regulatory acceptance, improve operator trust, and facilitate transparent integration of AI outputs into water-safety planning and compliance workflows. In safety-critical contexts such as drinking water, black-box predictions lack practical utility without interpretable reasoning. Approaches such as feature attribution, model-agnostic interpretation, and physics-informed ML can enhance transparency while improving robustness. Embedding hydraulic and transport constraints into data-driven models offers a pathway to reduce reliance on non-specific proxy variables and improve contamination specificity. Equally critical is ensuring model robustness under non-stationary conditions. Drinking water systems are inherently dynamic, influenced by temporal variability in demand, temperature, infrastructure integrity, and operational practices. Future systems must incorporate drift detection, adaptive learning, and continuous recalibration to maintain long-term reliability. Standardization represents a major research gap. The absence of open-access, high-quality datasets limits reproducibility and cross-system generalizability. Future efforts must prioritize standardized datasets incorporating not only water quality parameters but also metadata such as sensor calibration, maintenance history, and hydraulic context. Benchmarking frameworks must evolve to include temporal validation, cross-system transferability, and operational metrics such as detection latency and false alarm burden. Integration with public health surveillance systems is a critical translational frontier. Linking water quality data with health outcomes can enable early detection of disease outbreaks and support proactive risk management. Embedding AI outputs within water safety planning frameworks will ensure that anomaly detection translates into actionable interventions. Finally, scalability in low-resource settings must be prioritized. Affordable, modular systems combining low-cost sensors with lightweight models offer potential for decentralized monitoring. However, sustained impact requires field validation, capacity building, and institutional support. Future research must therefore adopt a systems-oriented approach, ensuring that AI contributes to measurable reductions in exposure and improved public health outcomes.

## 10 Conclusion

Wastewater contamination in drinking water systems remains a complex and persistent environmental health challenge that cannot be effectively addressed through conventional monitoring alone. This review establishes that contamination is not an isolated event but a pathway-driven, multi-barrier system failure occurring across interconnected components, including source water, treatment processes, distribution networks, and premise plumbing. Understanding this systemic nature is essential for identifying how contamination originates, propagates, and translates into human exposure. A key limitation of conventional monitoring lies in its temporal discontinuity, which creates a critical gap between contamination occurrence and detection. During this interval, exposure may already occur, particularly for microbial contaminants with low infectious doses. AI-based approaches offer potential to reduce this gap by supporting continuous monitoring, multivariate pattern recognition, and earlier identification of contamination-related anomalies. However, current evidence remains substantially stronger for predictive experimentation than for sustained operational deployment under real-world drinking water conditions. Their effectiveness, however, remains conditional and dependent on data quality, sensor reliability, validation rigor, and integration within operational frameworks. The synthesis of current AI methodologies highlights both promise and constraint. ML and DL models demonstrate strong predictive capabilities under controlled conditions, yet their real-world applicability is limited by reliance on non-specific proxy variables, inadequate temporal validation, and poor generalizability. Nevertheless, relatively few studies currently provide long-term field validation, cross-system transferability assessment, or integration with utility-scale public health response workflows.

These methodological limitations are further compounded by operational challenges such as sensor drift, data gaps, and the necessity of confirmatory testing. Consequently, AI should not be positioned as a standalone solution but as an augmentative component within a broader monitoring and response system. From a public health perspective, wastewater contamination presents significant risks, including acute microbial infections, chronic chemical exposures, and systemic inequities in water access and safety. Effective risk mitigation requires explicit linkage between detection systems and public health response mechanisms. AI can enhance this linkage by improving detection timeliness and enabling targeted interventions, but only when embedded within structured frameworks such as Water Safety Plan–based risk management. Ultimately, the value of AI in drinking water monitoring must be evaluated not by predictive accuracy alone, but by its ability to reduce detection latency, enable timely intervention, and measurably decrease population-level exposure risk. Achieving this requires a tightly integrated framework combining real-time sensing, AI-driven analytics, confirmatory validation, and decision-oriented governance. Future progress should therefore be judged based on demonstrable improvements in system resilience and public health protection, rather than incremental gains in model performance. From a policy and operational perspective, future implementation should prioritize standardized validation frameworks, integration of AI-enabled monitoring within Water Safety Plan–based risk management systems, and development of clear governance mechanisms for AI-assisted decision-making. Water utilities should combine AI-driven surveillance with confirmatory testing, operator training, and routine system maintenance to ensure reliable implementation. Policymakers and regulatory agencies should support the establishment of interoperable monitoring standards, data-sharing frameworks, and deployment guidelines that facilitate responsible adoption of AI technologies while maintaining public health protection as the primary objective. Accordingly, current AI applications in drinking water contamination monitoring should be interpreted as emerging decision-support tools with promising but still evolving operational maturity. Future research should prioritize temporal robustness, external validation, uncertainty quantification, and integration with operational water safety planning frameworks to enable reliable real-world implementation.
