# Correction: Pathway-driven assessment of wastewater contamination in drinking water systems: integrating AI with public health risk

**DOI:** 10.3389/fpubh.2026.1945614

**Published:** 2026-08-03

**Authors:** Frontiers Production Office

**Affiliations:** Frontiers Media SA, Lausanne, Switzerland

**Keywords:** anomaly detection, artificial intelligence, drinking water systems, machine learning, public health risk, sensor-based monitoring, wastewater contamination, water quality monitoring

An incorrect affiliation was erroneously assigned to reviewer Hemalatha Manupati. The correct affiliation is “KLEF Deemed to be University, India”. The affiliation has now been assigned.

The original version of this article has been updated.

